# Genomic assessment in *Lactobacillus plantarum* links the butyrogenic pathway with glutamine metabolism

**DOI:** 10.1038/s41598-017-16186-8

**Published:** 2017-11-21

**Authors:** Cristian Botta, Alberto Acquadro, Anna Greppi, Lorenzo Barchi, Marta Bertolino, Luca Cocolin, Kalliopi Rantsiou

**Affiliations:** 1 Department of Forestry, Agriculture and Food Sciences, University of Torino, Turin, Italy; 2 Present Address: Department of Health Sciences and Technology, Laboratory of Food Biotechnology, ETH Zürich, Switzerland

## Abstract

The butyrogenic capability of *Lactobacillus* (*L*.) *plantarum* is highly dependent on the substrate type and so far not assigned to any specific metabolic pathway. Accordingly, we compared three genomes of *L. plantarum* that showed a strain-specific capability to produce butyric acid in human cells growth media. Based on the genomic analysis, butyric acid production was attributed to the complementary activities of a medium-chain thioesterase and the fatty acid synthase of type two (FASII). However, the genomic islands of discrepancy observed between butyrogenic *L. plantarum* strains (S2T10D, S11T3E) and the non-butyrogenic strain O2T60C do not encompass genes of FASII, but several cassettes of genes related to sugar metabolism, bacteriocins, prophages and surface proteins. Interestingly, single amino acid substitutions predicted from SNPs analysis have highlighted deleterious mutations in key genes of glutamine metabolism in *L. plantarum* O2T60C, which corroborated well with the metabolic deficiency suffered by O2T60C in high-glutamine growth media and its consequent incapability to produce butyrate. In parallel, the increase of glutamine content induced the production of butyric acid by *L. plantarum* S2T10D. The present study reveals a previously undescribed metabolic route for butyric acid production in *L. plantarum*, and a potential involvement of the glutamine uptake in its regulation.

## Introduction

The genome-scale analysis of health-promoting bacteria is defined as probiogenomics and represents a fundamental approach to investigate their physiological behaviour or foresee potential probiotic and post-biotic features^1^. Among the intensively studied lactic acid bacteria (LAB) species, *Lactobacillus* (*L*.) *plantarum* is the most versatile one, and it is widely distributed in fermented dairy, sourdough, meat and vegetable foods^2,3^. *L. plantarum* is frequently encountered as a natural inhabitant of the human GastroIntestinal Tract (GIT), in which is a transient guest acquirable through the diet^4^, since it easily adapts its genome in response to the environmental niche requirements by acquiring, mixing or deleting several genomic-lifestyle islands that encode for specific metabolic activities^5,6^. Thus, *L. plantarum* genomic flexibility determines a broad range of phenotypes as well as strain-dependent beneficial features once it is introduced as probiotic in the diet, and consequently in the human GIT. Accordingly, the *L. plantarum* genomic data have been coupled with physiological observations to unravel the genetic determinants responsible for adhesion capability to the intestinal mucosa or immunomodulation of the host^7,8^. Together with the increasing knowledge over *L. plantarum* - host interactions, sophisticated bioinformatics tools have been developed using the reference strain *L. plantarum* WCFS1, including an advanced genome annotation^9^, genome-based metabolic models^10^, as well as effective mutagenesis tools^11^. However, despite those specific tools, there are still numerous uncharacterized pathways in *L. plantarum*, and they often encompass potential probiotic features.

The production of butyric acid is an example of a strain-dependent metabolic function often described in *L. plantarum* but, to best of our knowledge, not ascribed to any specific pathway at genomic level yet^12–15^. The impact of this short chain fatty acid (SCFA) on the intestinal homeostasis is well known, since it is capable to modulate the inflammatory status of the colon, colonic defense barrier, insulin sensitivity, intestinal epithelial permeability, oxidative stress, cryptic stem cells, colonic regulatory cells differentiation^16–19^ and, above all, it may act in the prevention and remediation of carcinogenesis^20,21^. In the human gut, butyric acid is the main end-product of intestinal microbial fermentation of undigested dietary fibers and its production is mainly ascribed to members of *Firmicutes*, such as *Lachnospiraceae*, *Ruminococcaceae* and *Clostridium* spp.^22^.

Accordingly, butyrogenic potential of any Human Intestinal Microbiome (HIM) can be currently determined by targeting the terminal genes of the main butyrate pathways, exploiting metagenomics or amplicon-based sequencing approaches^23^. These pools of terminal genes, encoding the conversion of butyryl-CoA to butyric acid, encompass several butyryl-CoA transferases (EC numbers: 2.8.3.8/2.8.3.9) and the butyrate kinase (2.7.2.7), which acts after the phosphorylation of butyryl-CoA^24,25^. Nevertheless, such approach may result reductionist, since it excludes the potential role of other butyrogenic metabolic pathway, such as the fatty acid metabolism, largely exploited by the industrial bioengineering of *Escherichia coli* ^26^.

In this context, we have shown in a parallel study how the putative probiotic strains *L. plantarum* O2T60C, S11T3E and S2T10D^27^ have potential anti-cancer activity in reason of a strain-specific butyrogenic capability expressed in a culture medium for human cell growth, known as Dulbecco’s Modified Eagle Medium (DMEM) (data not published). This medium represents a limited culture substrate for bacterial growth, lacking of recognized pro-butyrate substrates such as the fibers and mainly composed by glucose and glutamine^28^.

Therefore, the aim of this study was to associate, for the first time, the production of butyric acid in *L. plantarum* to a defined metabolic pathway. Moreover, we attempted to identify by functional and comparative genomics the potential genetic determinants and bioactive/growth substrates responsible for butyric acid strain-specific production in DMEM culture medium.

## Results

### Sequencing and comparative genomics reveals two distinct genotypes

The complete genomes of *L. plantarum* S2T10D, S11T3E and O2T60C were assembled in 92, 58 and 68 scaffolds respectively. Overall, the three *L. plantarum* strains showed genomes size ranging from 3.17 Mbp (strains S2T10D/S11T3E) to 3.31 Mbp for strain O2T60C (Table 1). Draft genomes were aligned to six reference *L. plantarum* genomes (WCFS1, P8, 16, JMD1, ZJ316 and ST-III) to calculate the pairwise genetic distances (data not shown). An average distance overall was calculated and resulted to be 0.00856. The scaffolds of the three strains were re-ordered using *L. plantarum* P8 strain (NC_021224.1) as guide reference, being the one with a genetic distance more similar to the average value, overall calculated. Both unplaced scaffold and putative plasmid genes were placed in the last position of the three *de novo* anchored genomes, generated by this ordering process. The reconstructed whole genome sequences of *L. plantarum* S2T10D, *L. plantarum* S11T3E and *L. plantarum* O2T60C have been deposited in the GenBank database, under the accession numbers MQNK00000000, MQNL00000000, MPLC00000000, respectively (Supplementary Table 1).

**Table 1:** General genomic features and comparative genomics of *L. plantarum* strains S2T10D, S11T3E and O2T60C, in comparison with the strain P8 (used as guide reference for the re-ordering of the scaffolds) and *L. plantarum* reference genome WCFS1.

	*L. plantarum* strains:	S2T10D	S11T3E	O2T60C	P8	WCFS1
**General genomic features**	Accession no.	MQNK00000000	MQNL00000000	MPLC00000000	NC_021224	NC_004567
	Genome size, Mbps	3.17	3.17	3.31	3.25	3.35
	N° scaffolds	92	58	68	8	4
	GC, content%	44.48	44.49	44.41	44.55	44.45
	No. of CDS	3,046	3,050	3,171	3,119	3,063
	tRNA genes	45	61	65	68	70
	rRNA genes (complete operons)	6 (1)	8 (1)	11 (1)	6 (4)	5 (5)
	Transposases	33	34	49	104	36
	Prophage clusters (intact)	3 (2)	3 (2)	4 (3)	2 (2)	3 (3)

All data reported are available at https://www.ncbi.nlm.nih.gov/.

The putative encoded proteomes vary in relation to the genome sizes (Table 1), harboring up to 3,000 proteins each one. InterProScan identified 2532 (S2T10D), 2546 (S11T3E) and 2660 (O2T60C) genes, with at least one domain (2917, 2971 and 3027 unique IPR domain for S2T10D, S11T3E and O2T60C, respectively). The top 20 SUPERFAMILY domains found in the three genomes, together with those harbored in the reference strain WCFS1 and the guide reference P8, are reported in Supplementary Figure 1. The most abundant protein superfamilies are P-loop containing nucleoside triphosphate hydrolase and “Winged helix” DNA-binding domains in accordance with what observed in the reference genomes of *L. plantarum* WCFS1 and P8. These domains mainly involve proteins acting in membrane transport (ABC transporter) and regulatory processes. Notably, the profile of O2T60C differs from those of S2T10D and S11T3E in the assignments of the three most abundant domains.

The relative distributions of COG categories in the three strains are similar to those of *L. plantarum* P8, while they are disproportionate compared to the reference genome of *L. plantarum* WCFS1^5^, which shows a greater number of proteins involved in the transport/metabolism of carbohydrates and a lower number of mobile genetic elements (Supplementary Figure 2). Concerning the mobile genetic elements, *L. plantarum* O2T60C possesses more transposase genes compared to S2T10D/S11T3E genomes, and also one additional intact phage cluster, identified by PHASTER tool as *Lactobacillus* phage Sha 1 (NC_019489). Noticeably, we did not identify CRISPRs motifs along the genomic sequences and only one single CRISPR-associated endonuclease Cas2 was annotated in the S2T10D/S11T3E genomes.

The OrthoMCL^29^ comparison performed among S2T10D, S11T3E and O2T60C clustered together a total of 8,168 sequences into 2,954 gene families (except singletons), highlighting a core-genome of 2,576 gene families. The same analysis conducted by comparing *L. plantatum* S2T10D, S11T3E and O2T60C with P-8 and WCSF1 strains proteomes, grouped a total of 13,655 sequences into 3,190 gene families (except singletons), highlighting a core-genome of 2,344 gene families (Supplementary Figure 3).

### Phylogenetic analysis

**Figure 1:**
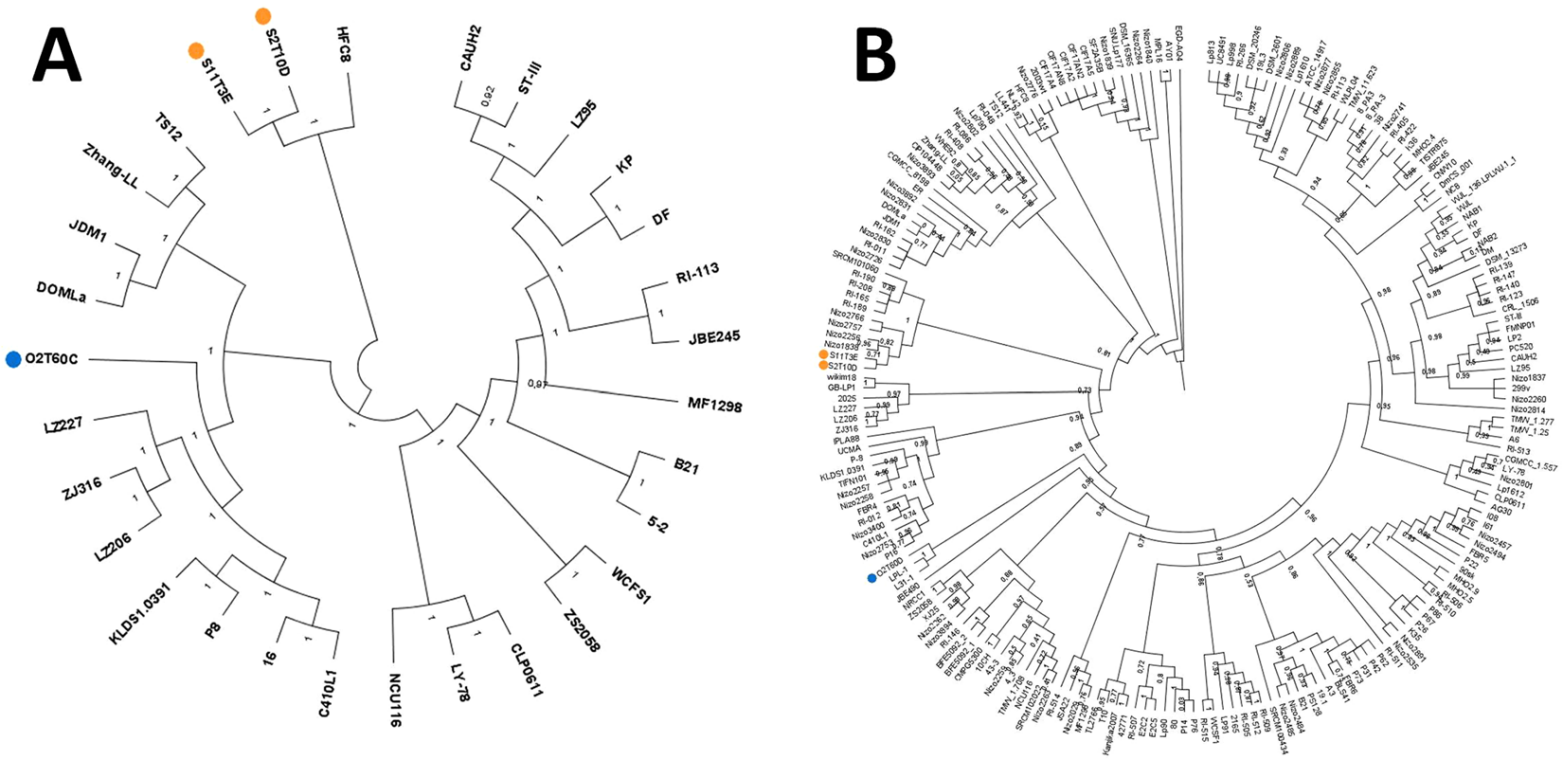
Phylogenetic trees including the genomes of the three *L. plantarum* analysed here and (**A**) other 27 complete genomes of *L. plantarum*, and (**B**) all *L. plantarum* genomes available (including both the complete as well as the unfinished sequences). The tree was constructed with Parsnp (https://github.com/marbl/parsnp), using the whole genome sequence of each selected strain. The strain O2T60C is highlighted by a blue circle, while S11T3E and O2T60C by orange ones. The bootstrap values are reported as a value ranging from 0 to 1.

A phylogenetic tree was constructed using nucleotide sequences of *L. plantarum* genomes, in order to highlight the genetic relation between the three strains with respect to 27 publicly available complete *L. plantarum* sequenced genomes (Supplementary Table 1). The analysis produced three well-separated clusters (Fig. 1A) and highlighted a pairwise high similarity of S2T10D and S11T3E, which clustered closed to the HCF8 strain. Strain O2T60C clustered in the clade with the LZ227 and LZ206, ZJ316, KLDS1.0391, P8 and P16 strains. All three strains were clearly separated from a third cluster, which included the reference WCFS1 and ST-III.

In addition a second tree was constructed using all the available *L. plantarum* sequenced genomes, thus including both the complete as well as the unfinished sequences. The analysis (Fig. 1B) highlighted the same high similarity of S2T10D and S11T3E, which clustered closed to some Nizo and RI strains. On the other side, strain O2T60C clustered in the clade with LPL-1 and L31-1 strains.

### Single nucleotide polymorphisms observed between O2T60C and butyrogenic strains

Assuming S2T10D/S11T3E and O2T60C as two different genotypes, we used the butyrate-producing strain S2T10D as backbone reference to analyze the number of single-nucleotide polymorphisms (SNPs) hosted in the core-genomes of the three strains (Table 2). Overall, we observed a high degree of sinteny between S2T10D and S11T3E, with only 15 nsSNPs detected in the genome of S11T3E and hosted by 4 genes (*lacL-lacM*; *brnQ*; BBA84_01095). However, PROVEAN analysis did not predict functional damages in the corresponding encoded proteins. The remaining SNPs retrieved in S11T3E generate synonymous mutation (90 SNPs) or are located in the intercistronic regions upstream or downstream the CDS (33 SNPs). On the other hand, 4,840 nsSNPs responsible of missense mutations in the amino acid sequences were detected in 1,598 genes of O2T60C.

**Table 2:** Cumulative summary and categorization of SNPs detected in the O2T60C and S11T3E genomes in comparison to the reference S2T10D.

S2T10D vs:	Synonimous SNPs	nsSNPs	High damage nsSNPs	non-coding SNPs	Total SNPs	No of genes hosting nsSNPS	No of genes hosting dSNPs
O2T60C	12464	4774	66	4544	21848	1598	64
S11T3E	90	15	0	33	138	4	0

Among the dSNPs found in the O2T60C, 66 mutations harbored in 64 genes generate deletion or addition of start/stop codons in each single host CDS, resulting in a functional damage of the encoded protein (Supplementary Table 2). The majority of the genes with high damage SNPs encoded for proteins with unknown function (11 genes), mobile elements (7 genes), proteins involved in the cell envelope biogenesis and outer membrane (11 genes).

### Overall gene content differences between O2T60C and butyrogenic strains

By combining the results from OrthoMCL analysis and KO assigned by KASS, we identified two sets of exclusive genes in the genotypes S2T10D/S11T3E and O2T60C, comprising 270 and 136 genes, respectively. Some of these were organized in genomic islands (GIs), which host in turn operons encoding for specific metabolic functions as well as proteins involved in bacteria-host or bacteria-environment interactions (Table 3). Overall, in O2T60C strain we observed unique set of genes that include a noticeable presence of phages, plasmid, transposases-related proteins (overall 43 genes) and elements related to DNA replication, recombination and repair (16 genes), with 43 and 16 genes respectively (Supplementary Table 3).

**Table 3:** Compositional features of the major genomic islands (GIs) of discrepancies observed between the genotypes O2T60C and S2T10D/S11T3E.

Genomes	GIs	Size (kb)	CDS Coordinates*	Overall functions
O2T60C	1	26.9	BBA85_00118 - BBA85_00148	Molybdopterin cofactor biosynthesis, iron transport, nitrite extrusion, kinase/response regulator system
	2	16.3	BBA85_01811 - BBA85_01827	Inositol uptake/metabolism and regulatory system, Galactilol PTS system
	3	13.1	BBA85_00355 - BBA85_00367	Galactilol PTS system
	4	8.2	BBA85_00391 - BBA85_00399	Rhamnose uptake/metabolism and regulatory system
	5	2.0	BBA85_02270 - BBA85_02472	Iron transport
S2T10D* S11T3E	6	9.1	BBF95_00141 - BBF95_00152	Conserved plantaricin operon
	7	7.9	BBF95_02519 - BBF95_02524	Type I restriction-modification system
	8	7.6	BBF95_00632 - BBF95_00638	EPS and CPS biosynthesis
	9	4.9	BBF95_01179 - BBF95_01183	EPS and CPS biosynthesis
	10	3.3	BBF95_01848 - BBF95_01846	Membrane proteins
	11	2.0	BBF95_02607 - BBF95_02609	Membrane proteins

Complete list of genes harbored exclusively in the two genotypes are reported in Supplementary Tables 2 and 3.

*CDS coordinates are referred to O2T60C and S2T10D.

Concerning the metabolic functions, only strain O2T60C possesses the complete operon *narGHJI*, encoding the nitrate reductase enzyme, and its molybdopterin cofactor biosynthesis genes (BBA85_00118 - BBA85_00148). The whole GI 1 enables the anaerobic respiration in *L. plantarum* by using nitrate and nitrite as electron acceptors^9^. Other GIs exclusive of O2T60C contain genes for the uptake and metabolism of specific sugars/alcohols, organized in gene cassettes encoding transporters, metabolic enzymes and regulatory proteins^6^. Among them, we annotated gene cassettes responsible of uptake and utilization of inositol (GI 2) and rhamnose (GI 4), though the latter gene cassette showed a truncation operated by transposases at the regulatory protein DeoR (BBA85_00399; BBA85_01826). Moreover, ABC-transporter of iron complexes (BBA85_02470 - BBA85_02472; BBA85_00118 - BBA85_00120) and the specific PTS systems for the galactitol uptake (BBA85_00358 - BBA85_00360; BBA85_01823 - BBA85_01825) were exclusively hosted in O2T60C genome.

The majority of genes shared by S2T10D and S11T3E encode for mobile genetic elements, for membrane and cell surface proteins, and for proteins with unknown functions, with 91, 43 and 37 genes respectively (Supplementary Table 4). Noticeable, the conserved loci organization of plantaricin regulon was found in this group of genes (GI 6). Moreover, this group of genes hosts two clusters of genes involved in capsular polysaccharides (CPS) and exopolysaccharides (EPS) biosynthesis (GIs 8 and 9), and also GIs encoding for membrane proteins (GIs 10 and 11).

### Identification of the metabolic route and triggering factors responsible of butyric acid production

#### Butyric acid is produced via fatty acid synthase of type II (FASII)

**Figure 2:**
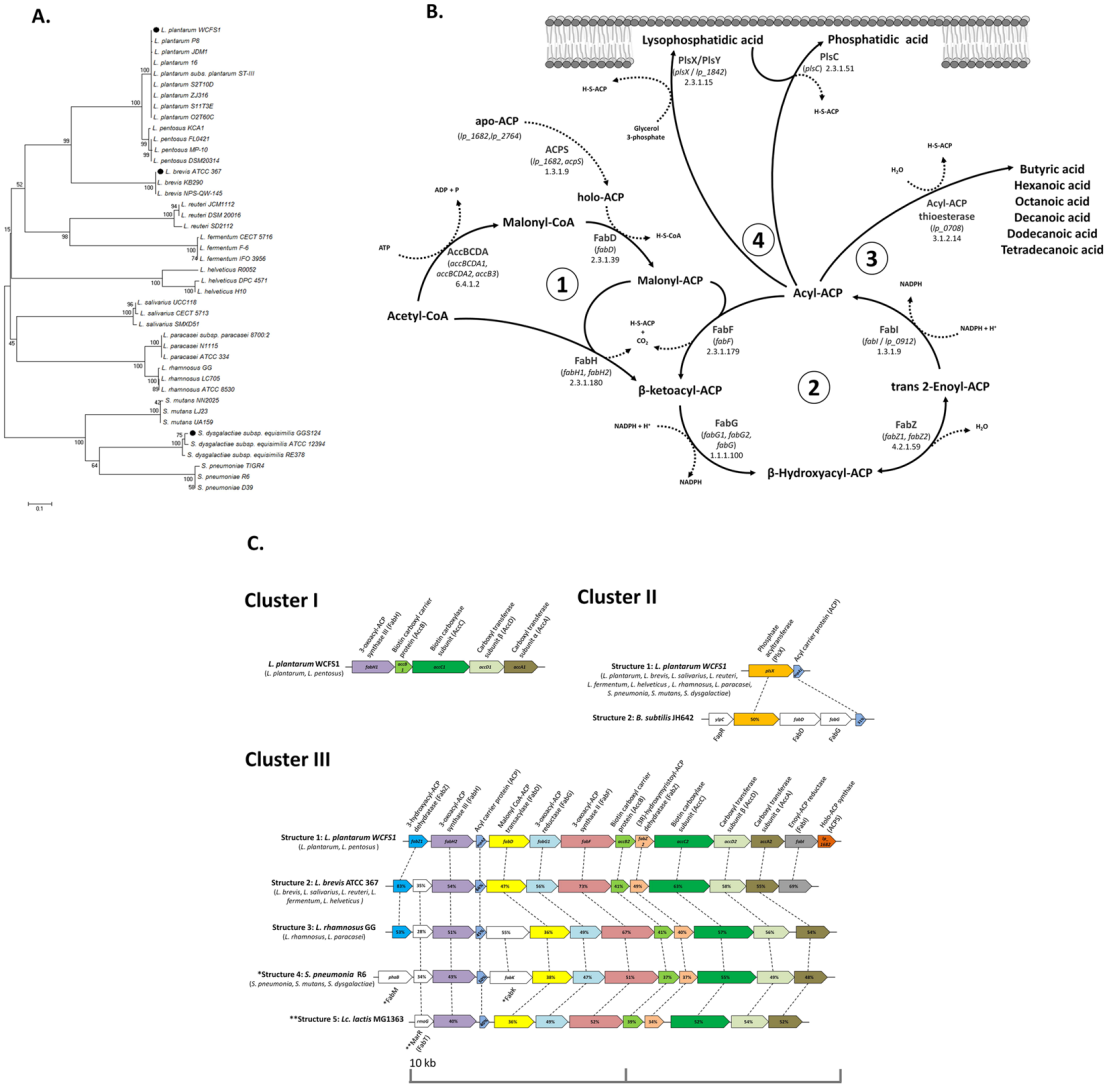
(**A**) Unrooted Neighbour-Joining tree based on the amino acid sequences of 43 acyl-ACP thioesterases (TEs) present in 13 gram positive bacterial species. TEs demonstrated capable to produce butyric acid are marked with black dots^30^. (**B**) Schematic representation of the conserved type II fatty acid biosynthesis pathway (FASII) and phosphate acyltransferase system (Pls) in *L. plantarum* species based on the reference strain *L. plantarum* WCFS1. Briefly, in the FASII initiation (1) the four separated subunits of acetyl-CoA carboxylase enzyme (Acc) catalyze the formation of malonyl-CoA starting from acetyl-CoA, with the biotin as covalently attached cofactor. Subsequently, the transacylase FabD substitutes the CoA for an acyl carrier protein (ACP), which is previously activated by phosphopantetheinyl-transferase (ACPS). The malonyl-ACP is condensed by FabH to acetyl-CoA, forming the β-ketoacyl-ACP. The β-ketoacyl-ACP enters in the iterative process of fatty acids chain-elongation (2), in which it is reduced by the FabG enzyme and dehydrated, first by FabZ (reversible reaction) and then by FabI (else FabK), producing the first four-carbons chain (C4) acyl-ACP. From this point the elongation process can continue through the dehydration of acyl-ACP carried out by FabF, or may be interrupted by a (3) medium-chain ACP-thioesterase (WP_003645113.), which cleaves the ester bonds and release free fatty acids with a chain length ranging from C4 and C14^30^. Once elongated up to C14^82^ the acyl-ACPs are transferred to the cells membrane (4) by acyltransferase (PlsX, PlsY, PlsC). (**C**) Genetic maps of FASII-Pls related genes in *L. plantarum* WCFS1 (representative loci organization of *L. plantarum* and *L. pentosus* species) and comparison with other Gram positive bacteria loci organizations, represented by the reference strains: *L. brevis* ATCC 367, *L. rhamnosus* GG, *S. pneumoniae* R6, *Lc. lactis* MG 1363 and *B. subtilis* JH 642. Species that have the same loci organization of each reference strain are reported in parentheses. Orthologous genes are denoted by the same color and connected by dashed lines while genes not present in *L. plantarum* are blanks. Proteins encoded are reported above or below the gene locus and the percentage of the amino acids similarity is reported for each homologous protein compared to *L. plantarum* WCFS1, or otherwise compared to a reference strain marked with asterisks (*).

Potential butyrogenic capability of isolates/sequenced genomes and metagenomes of whole communities are commonly inferred by targeting specific key genes that characterize the function, such as those encoding for the final enzymatic reaction in a butyrogenic pathway. As first step of analysis, we targeted the whole pool of terminal genes present in the acetyl-CoA/butyryl-CoA, ɣ-aminobutyrate/succinate, glutarate and lysine pathways, which represent the currently known butyrogenic metabolism of the HIM^24^. Practically, 521 amino acids sequences (http://img.jgi.doe.gov/) of the genes responsible for the enzymatic conversion of butyryl-CoA to butyric acid (EC numbers 2.8.3.8/2.8.3.9/2.7.2.7) were aligned to the predicted proteomes of S2T1D, S11T3E, O2T60C and the reference strain *L. plantarum* WCFS1. After the exclusion of these terminal genes and their respective butyrogenic pathways, we proceeded searching for all genes responsible of enzymatic reactions involved in the production of butyric acid (www.brenda-enzymes.org; http://www.genome.jp/kegg/annotation/enzyme.html; https://metacyc.org/). After this second step we identified in the medium-chain acyl-ACP thioesterase (lp_0708) the only possible terminal enzyme capable to produce butyric acid in *L. plantarum*. Indeed this enzyme, as well as those of *L. brevis* ATCC 367 and *S. dysgalactiae* subsp. *equisimilis* GGS 124, have been previously demonstrated capable to truncate the fatty acid biosynthesis pathway of type II (FASII) in engineered *E. coli*, releasing butyric acid and other medium chain fatty acids^30^. The amino acid sequences of the TEs belonging to 12 different species of Gram-positive bacteria were thus aligned. The Neighbor-Joining tree elaborated (Fig. 2A) has shown a high intra-specific homology among the amino acids sequences of TEs, and notably we did not observe for this enzyme any nsSNPs between the non-butyrogenic O2T60C and the strain S2T10D (Table 4).

**Table 4:** Complete list of proteins and encoding genes of *L. plantarum* S2T10D (butyrogenic strain) and *L. plantarum* O2T60C (non-butyrogenic strain) involved in the FASII-Pls pathway, together with results obtained from SNPs meaning (S2T10D vs. O2T60C) and prediction of the functional impact of mutation by means of PROVEAN.

Clusters	Proteins	Gene names*	E.C. no	Locus tag		O2T10D vs. S2T10D		
				S2T10D (butyrogenic)	O2T60C (non-butyrogenic)	nsSNPs	PROVEAN prediction (cutoff = −2.5)	Amino acids mutations (score)
**I**	3-oxoacyl-ACP synthase III (FabH)	*fabH1*	2.3.1.180	BBF95_00504	BBA85_01856	1	Neutral	
	Biotin carboxyl carrier protein (AccB)	*accB1*	6.4.1.2	BBF95_00505	BBA85_01857			
	Biotin carboxylase subunit (AccC)	*accC1*	6.4.1.2	BBF95_00506	BBA85_01858	3	Neutral	
	Carboxyl transferase subunit β (AccD)	*accD1*	6.4.1.2	BBF95_00507	BBA85_01859	2	Neutral	
	Carboxyl transferase subunit α (AccA)	*accA1*	6.4.1.2	BBF95_00508	BBA85_01860	3	Neutral	
**II**	Phosphate acyltransferase (PlsX)	*plsX*	2.3.1._	BBF95_00619	BBA85_01666			
	Acyl carrier protein (ACP)	*acpA1*	/	BBF95_00620	BBA85_01667			
**III**	3-hydroxyacyl-ACP dehydratase (FabZ)	*fabZ1*	4.2.1.59	BBF95_00945	BBA85_03004			
	3-oxoacyl-ACP synthase III (FabH)	*fabH2*	2.3.1.180	BBF95_00946	BBA85_03005			
	Acyl carrier protein (ACP)	*acpA2*	/	BBF95_00947	BBA85_03006			
	Malonyl CoA-ACP transacylase (FabD)	*fabD*	2.3.1.39	BBF95_00948	BBA85_03007	1	Neutral	
	3-oxoacyl-ACP reductase (FabG)	*fabG1*	1.1.1.100	BBF95_00949	BBA85_03008	1	Neutral	
	3-oxoacyl-ACP synthase II (FabF)	*fabF*	2.3.1.179	BBF95_00950	BBA85_03009			
	Biotin carboxyl carrier protein (AccB)	*accB2*	6.4.1.2	BBF95_00951	BBA85_03010			
	(3R)-hydroxymyristoyl-ACP dehydratase (FabZ)	*fabZ2*	4.2.1.59	BBF95_00952	BBA85_03011			
	Biotin carboxylase subunit (AccC)	*accC2*	6.3.4.14	BBF95_00953	BBA85_03012			
	Carboxyl transferase subunit β (AccD)	*accD2*	6.4.1.2	BBF95_00954	BBA85_03013	2	**Deleterious**	**Q231K (−3.255)**
	Carboxyl transferase subunit α (AccA)	*accA2*	6.4.1.2	BBF95_00955	BBA85_03014			
	Enoyl-ACP reductase (FabI)	*fabI*	1.3.1.9	BBF95_00956	BBA85_03015			
	Holo-ACP synthase (ACPS)	*acps*	2.7.8.7	BBF95_00957	BBA85_03016	1		
	Acyl-ACP thioesterase	lp_0708	3.1.2.14	BBF95_00554	BBA85_01904			
	Biotin carboxyl carrier protein (AccB)	*accB3*	6.4.1.2	BBF95_00112	BBA85_02896			
	3-oxoacyl-ACP reductase (FabG)	lp_0159	1.1.1.100	BBF95_01809	BBA85_02754			
	3-oxoacyl-ACP reductase (FabG)	*fabG2*	1.1.1.100	BBF95_01940	BBA85_00593			
	Putative enoyl-ACP reductase (FabI2)	lp_0912	1.3.1.9	BBF95_02498	BBA85_01269	2	Neutral	

*genes names are referred *to L. plantarum* WCFS1.

The FASII pathway located upstream the TE cleaving activity and the phosphate acyltransferase system (Pls) of *L. plantarum* were schematized in Fig. 2A. Moreover the *L. plantarum* FASII/Pls structure (i.e. loci organization) were compared with known structures of the 12 different species previously considered and the outgroup species *B. subtilis* JH 642 (Fig. 2C). Overall, this pathway in *L. plantarum* species encompasses 26 genes, of which 20 are organized in three operons, here named cluster I, II and III (Fig. 2B). The first cluster (I) harbors genes responsible of the FASII initiation and, among the species considered, is only detectable in *L. plantarum* and the closest *L. pentosus* species. Conversely, the structure present in the cluster II (PlsX-Acp) is highly conserved among the species considered, except for the outgroup *B. subtilis* JH 642 that hosts a thioesterase enzyme FapR, a transcriptional repressor of FASII and Pls genes^31,32^. The third and widest (~10 kb) cluster showed again a unique structure for *L. plantarum*/*L. pentosus* that notably lacks of FabT, a transcriptional repressor belonging to MarR family located upstream the 3-oxacyl-ACP synthase, which has been proven to repress the FASII operon in *Streptococcus spp*. and *Lactococcus lactis* and it may likewise act in *Lactobacillus spp*. that contain it in the same position^33–35^.

As far as the differences observed between S2T10D and O2T60C are concerned, all genes of this metabolic pathway are highly conserved while, in terms of mutations, the pathway of O2T60C harbors 16 nsSNPs compared to S2T10D. Notably, the mutation Q231K has been predicted deleterious (value of −3.255) for the functionality of the carboxyl transferase subunit β encoding gene located in the cluster III (Table 4).

#### Glutamine content triggers butyric acid production in DMEM culture media

In order to clarify the strain-dependent butyrogenic activity previously observed in high-glutamine supplemented DMEM (6 mM) we cultured the strains O2T6C and S2T10D in this human cells culture medium for 48 hours. In reason of the inhibitory activity of high amount of free amino acid versus lactobacilli^36^, we reduced the amount of glutamine to 2 mM. In parallel, we inoculated the strains in MRS and PBS, maintaining 0.45% of glucose as the only sugar available (Supplementary Table 5).

**Figure 3:**
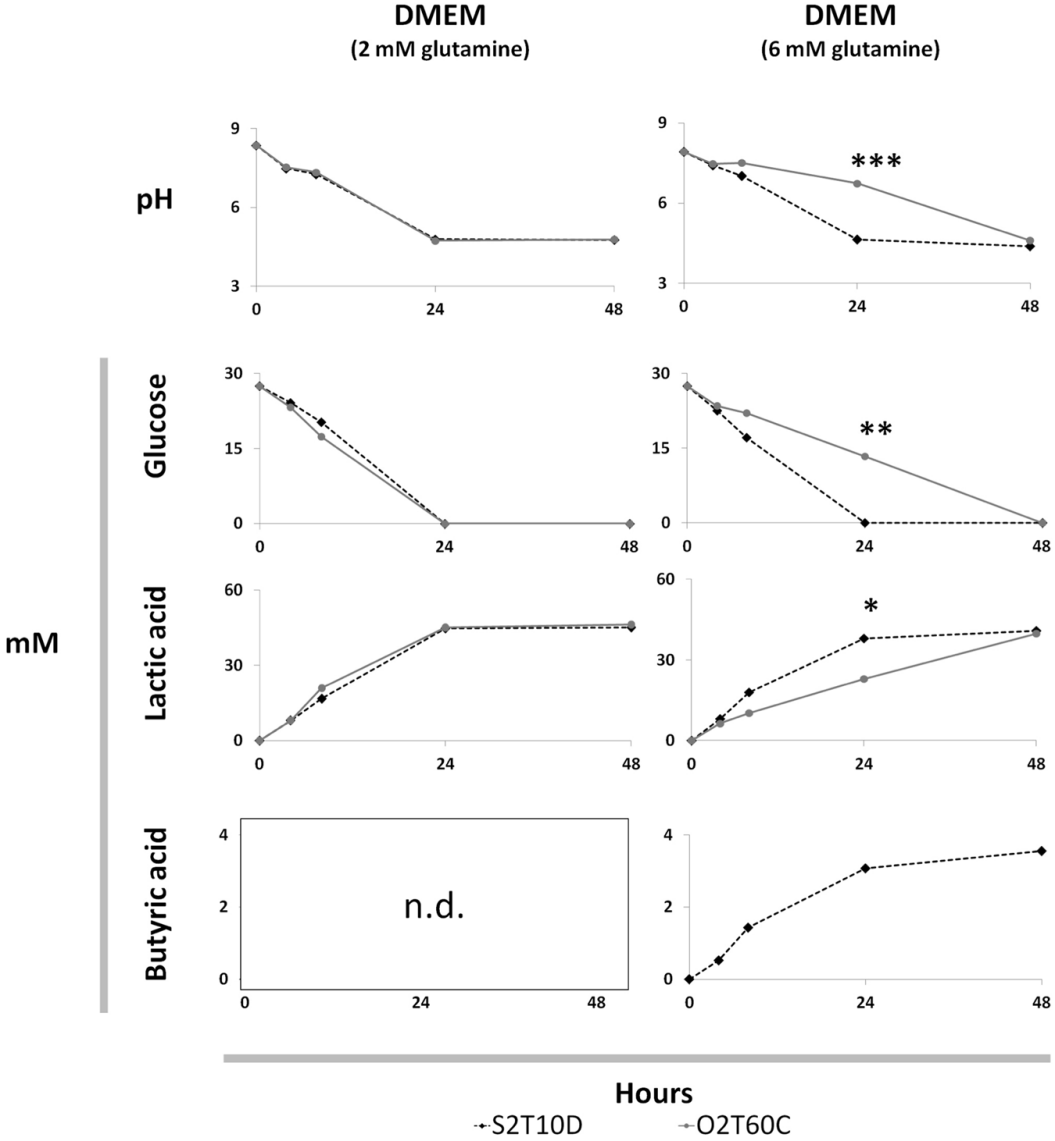
Variation of pH, glucose, lactic acid and butyric acid contents (mM) recorded during the fermentation of Dulbecco’s Modified Eagle’s Medium (DMEM; 0.45% of glucose) supplemented with 2 and 6 mM of L-glutamine and inoculated with *L. plantarum* O2T60C and S2T60C. The strains were inoculated at 8.0 ± 0.2 Log CFU and incubated for 48 h at 37 °C. Significant differences between the two strains O2T60C and S2T60D are highlighted with asterisk (T-test or Kolmogorov–Smirnov test; *p < 0.05; **p < 0.01; ***p < 0.001). Complete dataset of the four fermentation trials is reported in Supplementary Table 5 *n.d. = below the detection limit at all time points for both strains in the presence of 2 mM of L-glutamine.

The butyric acid was produced only by strain S2T0D once inoculated in the DMEM supplemented with 6 mM of glutamine. In both glutamine concentrations (2 and 6 mM) the O2T60C did not produce butyric acid at all, while in presence of 6 mM of glutamine it suffered a slowdown of the metabolic activities, compared to its behavior in 2 mM of glutamine and to the strain S2T10D dynamics (Fig. 3). Accordingly, in the 6 mM supplemented DMEM the consumption of glucose, lactic acid production and pH variation were significantly different between O2T60C and S2T10D the 24^th^ hour of incubation (*p* < 0.05). Finally, strains S2T10D and O2T60C showed the same metabolic behavior in MRS and glucose supplemented PBS.

**Figure 4:**
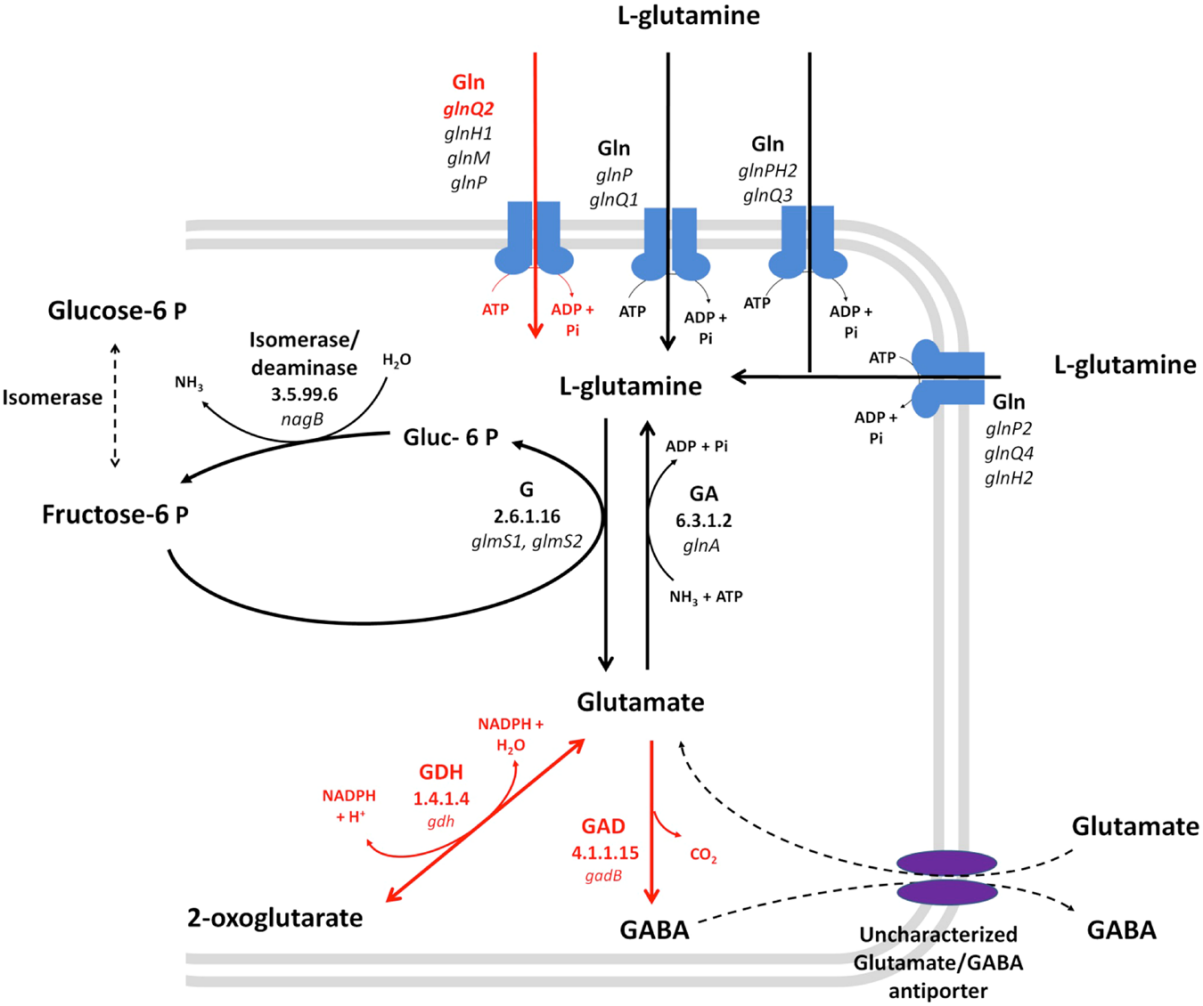
Schematic representation of glutamine uptake system and glutamine/glutamate central metabolism. Reconstruction was based on the reference strain WCFS1. Gln, Glutamine ABC-transporter; G, Glutamine–fructose-6-phosphate aminotransferase; GA, Glutamine synthetase; GDH, Glutamate dehydrogenase; GAD, Glutamate decarboxylase. Genes and relative enzymatic reactions labeled in red are predicted hosting deleterious mutations that may affect functionality (PROVEAN value below – 2.5). The complete list of reactions and genes of S2T10D and O2T60C involved in glutamine uptake and glutamine/glutamate metabolism is reported in Supplementary Table 6.

To elucidate the potential genomic-based causes behind the metabolic stress induced by glutamine in L*. plantarum* O2T60C, the O2T60C enzymes involved in all reactions encompassing glutamine and glutamate were compared to those of *L. plantarum* S2T10D (Supplementary Figure 6). Overall, O2T60C possess the same number of glutamine-related genes of S2T10D, for a total of 48. Within a pool of 86 nsSNPs observed in O2T60C, the central glutamine metabolism and the ABC transporter host three deleterious mutations responsible of a potential functional damage at enzymatic level. Indeed, we predicted deleterious functional mutations (PROVEAN score below – 2.5) for the amino acid sequences of *gnlQ2* gene (mutation P231S), encoding for a subunit of glutamine ABC transporter GlnQHMP, the glutamate dehydrogenase (GDH; mutation D408A) and the glutamate decarboxylase (GAD; V167A) (Fig. 4).

The subunit GlnQ2 of ABC transporter and GAD host deleterious mutations capable to affect their biological function with a high level of probability, with alignment-based scores of −7.602 and −3.806, respectively^37^. In accordance with the high level of probability that functional damage may occur, both deleterious mutations observed in GlnQHMP and GAD of O2T60C are not present in the six reference strains WCFS1, JMD1, ST-III, ZJ316, 16 and P8. Interestingly, the GAD is a singleton gene and, if confirmed, this predicted functional deficit cannot be remediated by other copy of the gene.

## Discussion

Comparative genomic analysis for *L. plantarum* strains S2T10D, S11T3E and O2T60C provided herein, confirms the high genomic flexibility and consequent physiological/metabolic versatility of this LAB species which can acquire, substitute or delete genomic regions and related metabolic features in response to the environmental niches^5^.

Overall, *L. plantarum* O2T60C was shown to be phylogenetically distant from strains S2T10D/S11T3E and to possess a distinct putative encoded proteome. Firstly, as expected, the genomic islands of divergence existing between O2T60C and S2T10D/S11T3E include mainly mobile genetic elements, such as transposases and prophage regions. Secondly, they include genomic regions known to be hyper-variable in *L. plantarum* strains isolated from different environments, and represented by gene clusters encoding for bacteriocins production, specific gene cassettes for carbohydrate and alcohol uptake/utilization, CPS/EPS biosynthesis genes and surface proteins^2,3^. It is noteworthy that O2T60C was isolated from the surface of olives in the final stages of fermentation, while strains S2T10D and S11T3E were recovered in fermentative brine at the early stages, which were previously characterized as two distinct ecological niches with differences in nutritional characteristics, such as types and amount of sugar available^38,39^.

As far as discrepancies in the metabolic activities, only O2T60C owns the complete genetic cluster enabling the heme-dependent anaerobic respiration^9,40,41^. The reduction of nitrate and nitrite to ammonia and NO is considered an exclusive metabolic feature of *L. plantarum* genome within the *Lactobacillus* genus, and once expressed it confers a strain-selective advantage during food fermentations. Anaerobic respiration may also increase the persistence in human GIT, where menaquinone is easily available and NO produced plays a role as signal molecule by which *L. plantarum* modulates physiology of GIT^41,42^. On the other hand, the group of genes exclusively hosted in *L. plantarum* S2T10D and S11T3E contain among others the conserved plantaricin regulon structure of *L. plantarum*, consisting of a regulatory operon (*plnABCD*), plantaricin EF operon (*plnEFI*) and the transport operon (*plnGHSTUVWX*)^43,44^. However, the regulon is devoid of PlnA pheromone and therefore not able to release any antimicrobial peptides^45^. These findings are in agreement with previous physiological tests, in which S2T10D and S11T3E did not inhibit pathogens by secretion of any proteinaceous compounds^27^. Regardless from lack of *plnA* gene, the whole operon *plnGHSTUVWX* has been demonstrated to be actively involved in the immune modulation of human dendritic cells, by acting on their inflammation status^7^. The presence and the potential expression of this transport operon in S2T10D/S11T3E, as well as the anaerobic respiratory capability of O2T60C deserve further investigation, to define whether these genomic features determine positive or negative impact in the GIT of the human host. Further, the group of genes absent or structurally damaged in the genome of O2T60C, contains several genes encoding for sortases, LPxTG-like cell wall anchor motif that have a pivotal role in bacterial intestinal colonization/adhesion^46,47^. However, *in vitro* experiments performed so far did not show significant and constant differences between adhesion capabilities of O2T60C and the other two strains^27^.

Besides the comparative genomics of the three strains, we also attributed the production of butyric acid to the complementary activities of the FASII pathway and the multispecies medium-chain acyl-ACP thioesterase (TE), enriching the current knowledge on the metabolic pathways reconstructed in *L. plantarum* ^5,9^. Despite the TE of *L. plantarum* WCFS1 has been demonstrated capable to produce butyric acid in engineered *E. coli* ^30^, so far the FASII-TE pathway has never been indicated as the only butyrogenic pathway present in this species. Aligning the TEs of different Gram-positive bacteria, we observed homologies in accordance with the overall phylogenetic distance of the species^48,49^. Notably, within *L. plantarum* species the TEs have shown the same amino acid sequences and hence they potentially express the same potentially butyrogenic function. This observation seemed to indicate the upstream FASII pathway as the likely responsible of modulating the butyric production in S2T10D and O2T60C. However, we predicted in the FASII pathway of O2T60C only a single deleterious mutation for the carboxyl transferase subunit β (*accD2*), which, in reason of the redundancy of this gene, may not determine any severe consequence for the FASII functionality.

Comparing the FASII pathway of different Gram-positive species, we observe a peculiar structure and loci organization of *L. plantarum* and *L. pentosus*, which lacks both transcriptional regulators FabT and FapR, respectively involved in the FASII repression of LAB and Gram-positive pathogens like *Bacillus subtilis* ^31–35^. The absence of these FASII repressors in *L. plantarum*/*L. pentosus* enables us to hypothesize a major role of TE enzymes in the modulation of lipids metabolism and cell membrane structure, by interrupting the fatty acid elongation process in response to external stimuli. Overall, the FASII pathway plays a central role in the adaptation of Gram-positive bacteria to external environment since, in response to nutritional factors available and physic-chemical condition, the cell membrane composition is selectively modified by complex regulatory networks of genes^50,51^. For instance, the fructooligosaccharides (FOS) has recently been demonstrated capable of altering the membrane fluidity of *L. plantarum*, acting on the FASII transcriptional patterns^52^. The same FOS, and the inulin, seem to significantly trigger the butyrogenic capability of *L. plantarum* ^12,13^, in accordance with the frequent association between fibers uptake and SCFA produced by the human intestinal microbiota in large intestine^53^.

However, in our specific case DMEM, like all human cells culture media, lacks of these prebiotics and it is mainly composed by glucose and glutamine. Notably, by increasing the glutamine content from 2 to 6 mM we observed a slowdown in the metabolic activities for the strain O2T60C, while in parallel, the production of butyric acid was elicited in S2T10D. To this regard, the glutamine has recently been correlated with an enhanced production of butyric acid by intestinal microbiota and a modulation of *Lactobacillus* populations in mice dietary supplemented with *L. plantarum* ^54,55^. Interestingly, in the genome of O2T60C three functional mutations were predicted in the glutamine uptake system and its metabolism, which play a central role in the regulation of amino acids catabolism of LAB^36,56^. The potential dysfunction of the ABC-transporter GlnQHMP cannot significantly impact the uptake of glutamine in reason of the redundancy of these ABC-transporter^9^. On the other hand, GDH and GAD enzymes are encoded by singletons and are responsible of dehydrogenation and decarboxylation of glutamate respectively. Their potential functional damage may result in a limited cell resistance of the *L. plantarum* in response to external stimuli, such as low pH^57–59^. Despite we found an effective correlation between physiological behaviours and SNPs analysis, the regulatory network by which the glutamine induces in parallel the butyric acid production in S2T10D and limit the growth of O2T60C, remains beyond the potentiality of this first comparative genomic study, which however provides strong bases for guiding further omics investigations.

In summary, we identified and characterized for the first time the FASII-TE pathway as the only metabolic route responsible of butyric acid production in *L. plantarum* species, whereas in parallel we observed in our strains S2T10D and O2T60C a clear involvement of the glutamine in its production.

## Materials and Methods

### DNA sequencing and genome reconstruction

The genome sequences of *L. plantarum* strains S2T10D, S11T3E and O2T60C were determined by GenProbio SRL (Parma, Italy) using the Ion Torrent Personal Genome Machine (PGM; Life Technologies, USA). Briefly, a genomic library was generated using 10 µg of genomic DNA and an Ion Xpress Plus fragment library kit and employing the Ion Shear chemistry according to the user guide. After dilutions, molecules were used as the templates for clonal amplification on Ion Sphere particles during the emulsion PCR according to the Ion PGM template 400 kit manual. DNA quantitation was performed via library quantitation of DNA standards (Kapa Biosystems). The quality of the amplification was estimated, and the sample was loaded onto an Ion 316 chip and subsequently sequenced using 212 sequencing cycles according to the Ion PGM sequencing 400 kit user guide. This number of sequencing cycles resulted in an average reading length of approximately 400 nucleotides.

Raw reads were analyzed with Scythe (https://github.com/vsbuffalo/scythe) for filtering out contaminant substrings and Sickle (https://github.com/najoshi/sickle), which allows to remove reads with poor quality ends (Q < 30). *De novo* assembly was performed with the Mira (version 3.4.0) assembler^60^. Contigs were manually inspected for errors and chimeric contigs, due to overlapping mobile elements, were split. Genome reconstruction of each strain was performed submitting the assembled sequences to the Mauve suite (https://www.sourceware.org/mauve). This kind of reconstruction is a reference guided-ordering of the scaffolds based on iterative alignment steps using a known genome. It does not reconstruct a unique chromosome, but it produced a multi-fasta file with a high-confident scaffold order. To select the best reference genome for the reconstruction, all the publicly available sequenced *L. plantarum* genomes were, at first, compared with the 3 strains and pairwise genetic similarity values were recorded using the genome-to-genome distance calculator (GGDC 2.0: http://ggdc.dsmz.de/distcalc2.php).

### Genome annotation and OrthoMCL analysis

Genomes were structurally annotated using the PROKKA suite^61^, which uses a collection of annotation procedures (e.g.: Prodigal), to generate coding sequences (CDS), proteins and.gbk files for each analyzed strain. Gene functions were assigned to predicted genes using the HMMER suite (v3.1, http://hmmer.org) adopting the TREMBL bacteria database as refs^62^, with default parameters, with the exception of sequence E-value (e^−10^) as well as with InterproScan^63^ (ver. 5.18-57.0;) against all the available databases (ProSiteProfiles-20.119)^64^, PANTHER-10.0^65^, Coils-2.2.1^66^, PIRSF-3.01^67^, Hamap-201511.02^68^, Pfam-29.0^69^, ProSitePatterns-20.119^64^, SUPERFAMILY-1.75^70^, ProDom-2006.1^71^, SMART-7.1^72^, Gene3D-3.5.0^73^ and TIGRFAM-15.0^74^. Additional information on metabolic pathways was highlighted using the KASS tool^75^, adopting the bidirectional best-hit default mode, against all known enzymes of *L. plantarum* species. COG (Clusters of Orthologous Groups) annotation was conducted using WebMGA^76^. Prediction of phage clusters was carried out with PHASTER^77^.

The annotated proteomes of S2T10D, S11T3E and O2T60C were used to conduct an OrthoMCL^29^ analysis for identifying common and distinctive orthologues sets. In addition, an OrthoMCL analysis was conducted on S2T10D, S11T3E and O2T60C with P8 and JDM1 strains proteomes.

### Phylogenetic analysis

Phylogenetic analysis of the *L. plantarum* strains was conducted using Parsnp^78^, a rapid core genome multi-alignment algorithm freely available (https://github.com/marbl/parsnp). The genome sequences of 27 fully assembled *L. plantarum* strains were downloaded from NCBI (https://www.ncbi.nlm.nih.gov/genomes/MICROBES/microbial_taxtree.html) and aligned together with O2T60D, S11T3E and S2T10D, using the WCFS1 strain as reference (Fig. 1). The phylogenetic tree was generated using FigTree (http://tree.bio.ed.ac.uk/software/figtree).

### Analysis of single nucleotides polymorphisms (SNPs)

Raw reads of the strains O2T60D and S11T3E were back-aligned to the S2T10D genome, which was selected as the reference in relation to the comparative analysis and its butyrogenic capability in DMEM. The alignment was performed with Burrows-Wheeler Aligner program (BWA^79^) and ‘mem’ command, with default parameters. The BAM files were processed and adapted for SNP calling program with SAMtools mpileup^80^, using default parameters with the exclusion of minimum mapping quality equal to 25 and filtering ambiguous read mapping. Results were filtered taking into account two parameters: the SNPs call quality and depth. SNPs having mapping quality lower than 20 were removed. In addition we set as lower limit of mapping depth a value of 8 and the upper limit was set to 200, 70 and 50 for O2T60C, S11T3E and S2T10D, respectively.

Identified variants of O2T60C and S11T3E compared to S2T10D were analyzed using SNPeff (http://snpeff.sourceforge.net/) and classified into four classes: (i) non-coding SNPs, for the variants located outside the CDS; (ii) synonymous SNPs, for variants in CDS, which do not modify the amino acid sequence, (iii) non-synonymous SNPs (nsSNPs), for variants in CDS, which modify amino acid sequence and (iv) deleterious SNPs (dSNPs) which generate, frameshifts, gaining or loss of stop and start codons, causing functional deleterious mutations in the encoded proteins^81^. The nsSNPs, in genes belonging to pathways of interest, were also analyzed with PROVEAN (Protein Variation Effect Analyzer algorithm), which predicts the functional impact for all classes of protein sequence variation, such a single amino acid substitution, insertion, deletion and multiple substitution. The score threshold used was set to −2.5^37^.

### Research of candidate genes responsible of butyric acid production in *L. plantarum* species

A total of 521 protein sequences belonging to butyrate kinase (Buk), butyryl-CoA transferase (But, AtoA, Atod, 4Hbt) and alternative –CoA transferase were acquired from metagenomic database (http://img.jgi.doe.gov/) of butanoate pathways present in bacteria^24^ and aligned with the genomes of S2T10D, S11T3E, O2T60C and the reference *L. plantarum* WCFS1 by BlastP, using default parameters. Candidate orthologous genes were selected considering a minimum of 30% identity over at least 80% of both protein lengths, as filtering threshold. For each of the searched genes (521), if the blastP produced an under threshold score, the orthologous protein was considered absent from the analyzed genome.

Moreover, the presence of all known genes responsible of butyric acid production were manually checked in the genomes by referring to the main public available databases (www.brenda-enzymes.org; http://www.genome.jp/kegg/annotation/enzyme.html; https://metacyc.org/).

### Growth dynamics in different substrates

All materials were provided from Sigma-Aldrich (Saint Louis, MO, USA), unless otherwise stated. The *L. plantarum* strains were routinely cultivated in Man Rogosa Sharpe (MRS) and Brain Heart Infusion (BHI) culture broths (Lab M, Heywood, Lancashire, UK). Stock bacterial cultures were kept at −80 °C with 20% of glycerol. Before performing the physiological tests, a single fresh colony of each bacterial strain was inoculated in the appropriate culture broth, grown overnight and then added at ratio 1:100 in new fresh broth. This suspension was grown until the bacteria reached early-stationary phase (18 h), and thus used for the physiological tests (working culture). The initial concentration of each working culture was determined by optical density (OD) at 630 nm with ELx880 microtiter plate reader (Savatec, Turin, Italy). All the bacterial suspensions were set to the same initial count using an internal standard curve.

The working culture of each strain was washed twice in Ringer’s solution and inoculated at 8.0 ± 0.2 Log CFU mL^−1^ in four different culture broths: (A) Phosphate Buffer Saline (PBS) with 0.45% of glucose; (B) Dulbecco’s Modified Eagle’s Medium (DMEM) supplemented with 2 mM of L-glutamine and 0.45% of glucose; (C) Dulbecco’s Modified Eagle’s Medium (DMEM) supplemented with 6 mM of L-glutamine and 0.45% of glucose; (D) modified MRS (mMRS) medium (10 g L^−1^ of bacteriological peptone; 8 g L^−1^ of soy peptone; 10 g L^−1^ of yeast extract; 1 mL L^−1^ of tween-80; 2 g L^−1^ of K_2_PO_4_; 2 g L^−1^ of triammonium citrate; 0.2 g L^−1^ of MgSO_4_) with 0.45% of glucose as only sugar available.

Inoculated culture broths (40 mL) were incubated in 50 mL centrifuge tube for 48 hours at 37 °C, with periodic shaking. Samples (4 mL) were taken after 4 h (early exponential phase), 8 h (exponential phase), 24 h (stationary phase) and 48 h (late stationary/decline phase), and the microbiological enumeration was performed by CFU method. Samples were centrifuged (14 000 × g for 10 minutes), filtered (0.2 µm cellulose acetate) and cell free supernantants (CFS) were kept at −20 °C until the analysis of organic acids and sugars were performed. CFS pH was measured at each sampling point using a pH meter (Crison, Modena, Italy).

### HPLC analysis

Organic acids (citric, pyruvic, lactic, acetic, butyric and propionic) and sugars (lactose, glucose and galactose) were determined by high performance liquid chromatography (HPLC). The HPLC system (Thermo Finnigan Spectra System, San Jose, USA) was equipped with an isocratic pump (P4000), a multiple autosampler (AS3000) fitted with a 20 µL loop, a UV detector (UV100) set at 210 and a refractive index detector RI-150. For the organic acid the analyses were performed isocratically, at 0.8 ml min^−1^ of a 0.013 N H_2_SO_4_ as mobile phase at 60 °C, with a 300 × 7.8 mm i.d.cation exchange column (Aminex HPX-87H) at 60 °C equipped with a Cation H+ Microguard cartridge (Bio-Rad Laboratories, Hercules, USA). For the sugars, the analyses were performed isocratically, at 1 ml min^−1^ of H_2_O as mobile phase, with a 300 × 7.8 mm i.d.cation exchange column (Aminex HPX-87P) at 60 °C equipped with a Carbo-P Microguard cartridge (Bio-Rad Laboratories, Hercules, USA). The data treatments were carried out using the Chrom QuestTM chromatography data system (ThermoQuest Corporation, San Jose, USA). Analytical grade reagents (Sigma-Aldrich, St. Louis, USA) were used.

### Data availability statement

The data reported in the manuscript are publically available.

## Electronic supplementary material

Supplementary material
