# Supplementary material for: Genomic assessment in Lactobacillus plantarum links the butyrogenic pathway with glutamine metabolism
Source: Sci Rep. 2017 Nov 21;7:15975. doi: 10.1038/s41598-017-16186-8 (PMC5698307; doi:10.1038/s41598-017-16186-8)
Supplement: Supplementary file 1 — Supplementary material [file 41598_2017_16186_MOESM1_ESM.pdf]

Genomic assessment in *Lactobacillus plantarum* links the butyrogenic pathway with glutamine metabolism

Cristian Botta[1], Alberto Acquadro[1], Anna Greppi[1,$,] Lorenzo Barchi[1], Marta Bertolino[1], Luca Cocolin[1], Kalliopi Rantsiou[1]

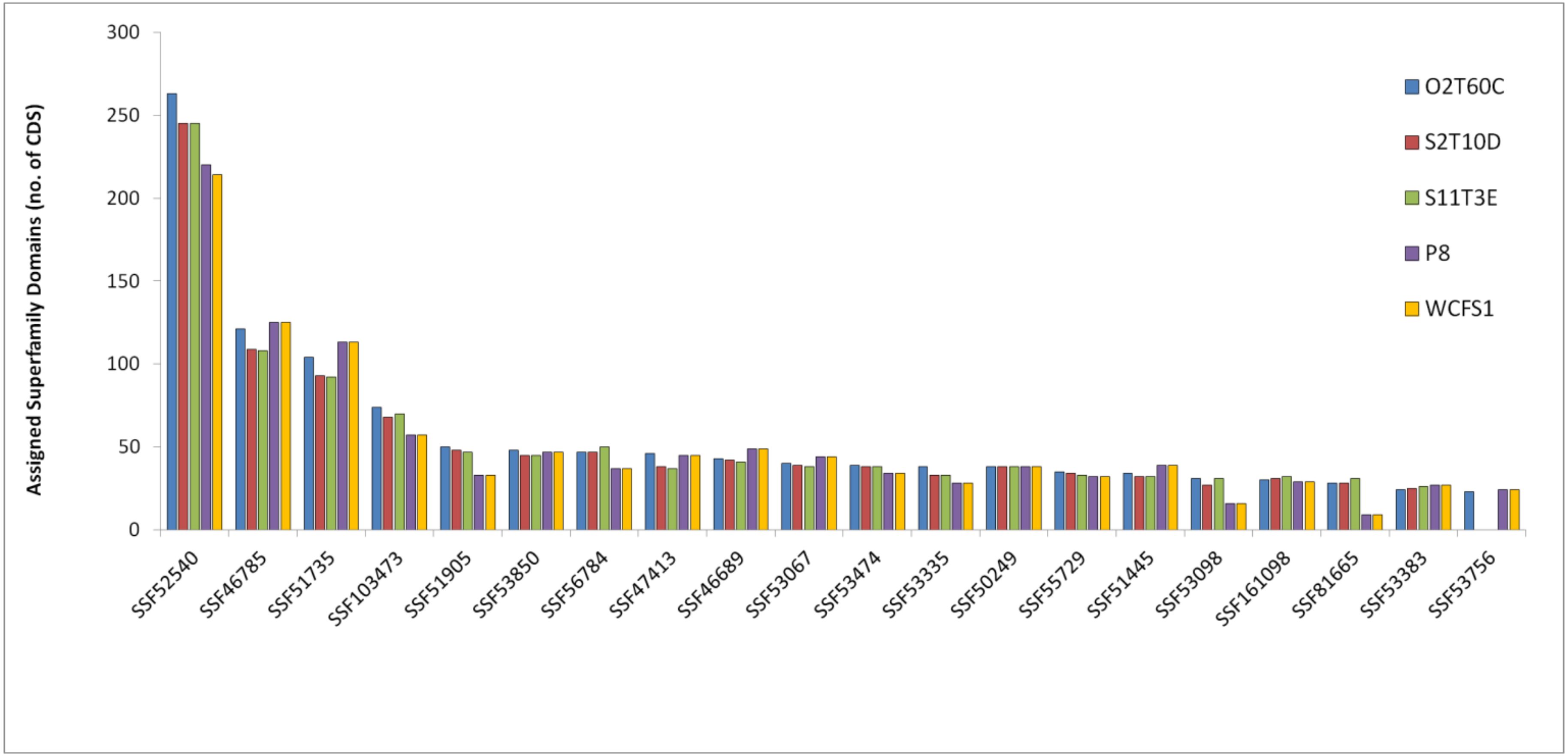

Supplementary Figure 1.

Top 20 proteins superfamily domains identified in the three genomes (O2T60C, S2T10D and S11T3E) annotated using Interproscan pipeline, and comparison with reference *L. plantarum* genomes P8 and WCFS1 (http://supfam.org/SUPERFAMILY/index.html). Superfamily domains were listed in decreasing order: P-loop containing nucleoside triphosphate hydrolases (SSF52540); "Winged helix" DNA-binding domain (SSF46785); NAD(P)-binding Rossmann-fold domains (SSF51735); MFS general substrate transporter (SSF103473); FAD/NAD(P)-binding domain (SSF51905); Periplasmic binding protein-like II (SSF53850); HAD-like (SSF56784); lambda repressor-like DNA-binding domains (SSF47413); Homeodomain-like (SSF46689); Actin-like ATPase domain (SSF53067); alpha/beta-Hydrolases (SSF53474); S-adenosyl-L-methionine-dependent methyltransferases (SSF53335); Nucleic acid-binding proteins (SSF50249); Acyl-CoA N-acyltransferases (Nat) (SSF55729); (Trans)glycosidases (SSF51445); Ribonuclease H-like (SSF53098); MetI-like (SSF161098); Calcium ATPase, transmembrane domain M (SSF81665); PLP-dependent transferases (SSF53383); UDP-Glycosyltransferase/glycogen phosphorylase (SSF53756).

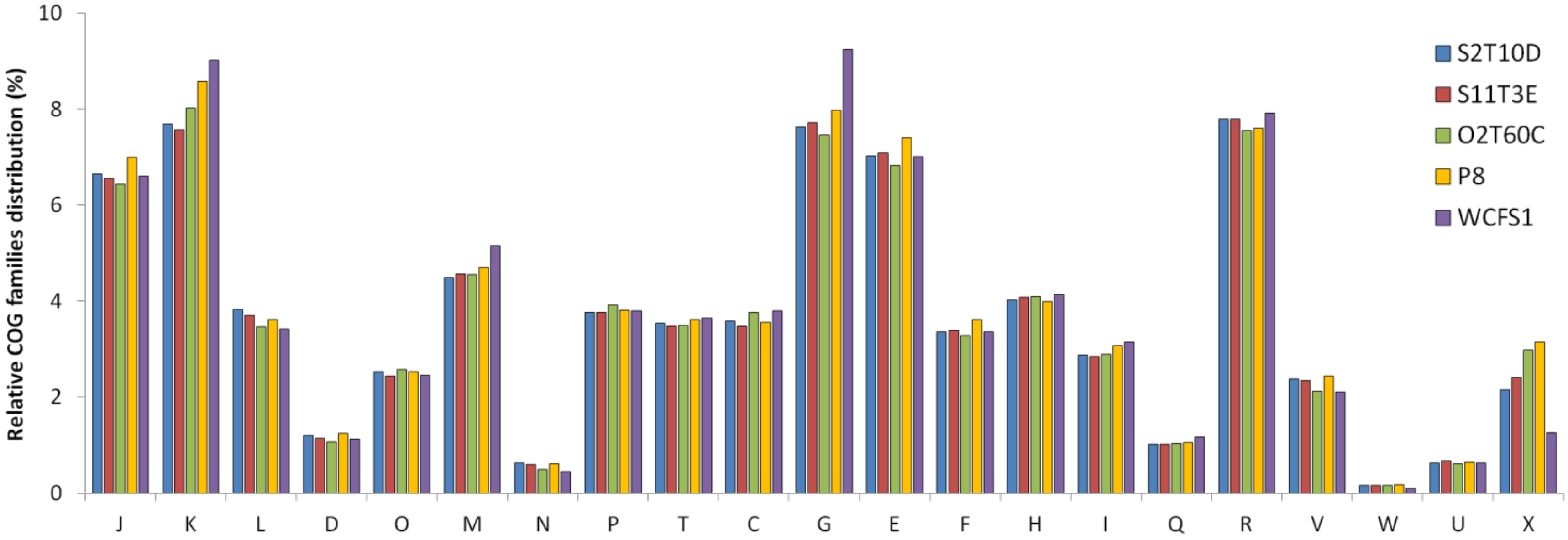

Supplementary Figure 2.

Distribution of COG categories in the *L. plantarum* strains S2T10D, S11T3E, O2T60C, P8, WCFS1. COG categories were listed in the following order: translation, ribosomal structure and biogenesis (J); transcription (K); DNA replication, recombination, and repair (L); cell division and chromosome partitioning (D); post-translational modification, protein turnover, chaperones (O); cell envelope biogenesis, outer membrane (M); cell motility (N); inorganic ion transport and metabolism (P); signal transduction mechanisms (T);energy production and conversion (C); carbohydrate transport and metabolism (G); amino acid transport and metabolism (E); nucleotide transport and metabolism (F); coenzyme metabolism (H); lipid metabolism (I); secondary metabolites biosynthesis, transport, and catabolism (Q); general function prediction only (R); defense mechanisms (V); extracellular structures (W); intracellular trafficking, secretion, and vesicular transport (U); mobilome: prophages, transposons (X).

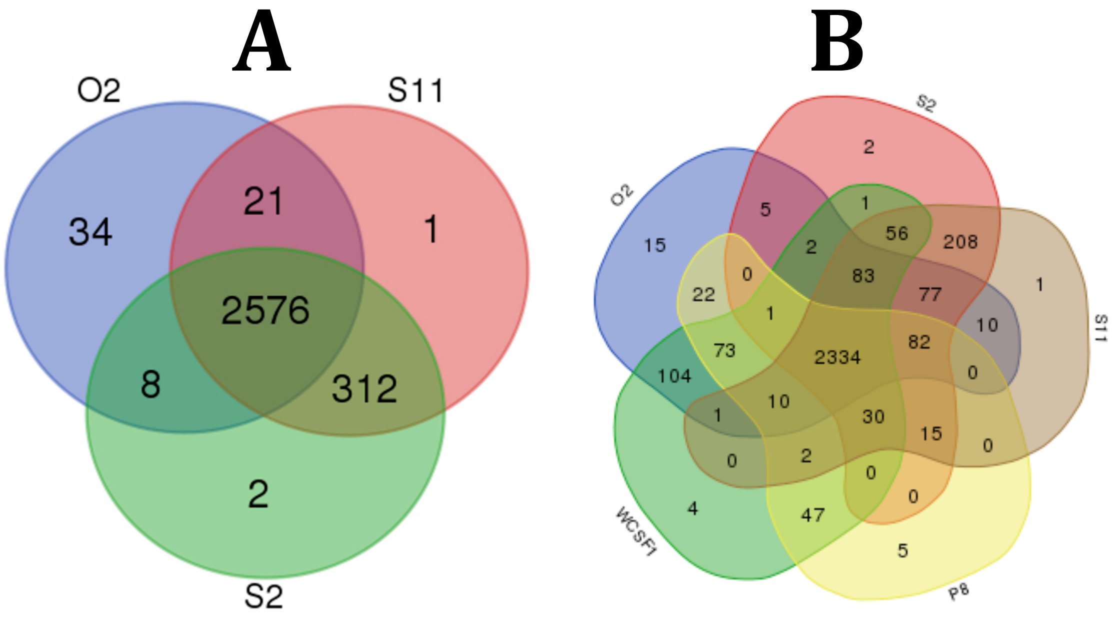

Supplementary Figure 3.
Venn diagram of OrthoMCL[1] analyses on S2T10D (S2), S11T3E (S11) and O2T60C (O2) proteomes (A) as well as on S2, S11, O2, P-8 and WCFS1 proteomes (B).

Supplementary Table 1. List of bacterial genomes used in this study and accession numbers.

**Genus**	**Species**	**Subspecies**	**Strain**	**Accession no.**
Lactobacillus	*plantarum*	*plantarum*	WCFS1	NC_004567.2
			P-8	NC_021224.2
			JMD1	NC_012984.1
			ST-III	NC_014554.1
			ZJ316	NC_020229.1
			16	NC_021514.1
			B21	NZ_CP010528.1
			C410L1	NZ_CP017954.1
			CAUH2	NZ_CP015126.1
			DF	NZ_CP013753.1
			DOMLa	NZ_CP004406.1
			HFC8	NZ_CP012650.1
			JBE245	NZ_CP014780.1
			KP	NZ_CP013749.1
			LY-78	NZ_CP015308.1
			LZ95	NZ_CP012122.1
			LZ206	NZ_CP015966.1
			LZ227	NZ_CP015857.1
			MF1298	NZ_CP013149.1
			NCU116	NZ_CP016071.1
			TS12	NZ_CP018324.1
			Zhang-LL	NZ_CP011769.1
			ZS058	NZ_CP012343.1
			5-2	NZ_CP009236.1
			RI-113	NZ_CP017407.1
			CLP0611	NZ_CP019722.1
			KLDS1.0391	CP019348.1
	pentosus		KCA1	NZ_CM001538.1
			MP-10	NZ_FLYG00000000.1
			FL0421	NZ_LFLY00000000.1
			DSM20314	NZ_AZCU00000000.1
	fermentum		IFO 3956	NC_010610.1
			CECT 5716	NC_017465.1
			F-6	NC_021235.1
	helveticus		DPC 4571	NC_010080.1
			H10	NC_017467.1
			R0052	NC_018528.1
	paracasei		ATCC 334	NC_008526.1
			8700:2	NC_022112.1
			N1115	NZ_CP007122.1
	reuteri		DSM 20016	NC_009513.1
			JCM1112	NC_010609.1
			SD2112	NC_015697.1
	rhamnosus		GG	NC_013198.1
			LC705	NC_013199.1
			ATCC 8530	NC_017491.1
	brevis		ATCC 367	NC_008497.1
			KB290	NC_020819.1
			NPS-QW-145	NZ_CP015398.1
	salivarius		UCC118	NC_007929.1
			CECT 5713	NC_017481.1
			JCM1046	NZ_CP007646.1
Streptococcus	*dysgalactiae*	*equisimilis*	GGS_124	NC_012891.1
			ATCC 12394	NC_017567.1
			RE378	NC_018712.1
	pneumoniae		R6	NC_003098.1
			D39	NC_008533.1
			TIGR4	NC_003028.3
	mutans		UA159	NC_004350.2
			NN2025	NC_013928.1
			LJ23	NC_017768.1
Lactococcus	*lactis*	*cremoris*	MG1363	NC_009004.1
Bacillus	*subtilis*	*subtilis*	JH642 (str. 168)	NC_000964.3

Supplementary Table 2. Genes of O2T60C hosting nsSNPs which determine a gain of start and stop codons, or deletion of stop codons.

	Locus tag S2T10D					SNPs (nucleotide substitutions)		
**COG categories**	**S2T10D**	**O2T60C**	**Encoded proteins**	**KO**	**EC number**	**Start lost**	**Stop gained**	**Stop lost**
Carbohydrate transport and metabolism	BBF95_00309	BBA85_01907	Phosphonoacetaldehyde hydrolase	K05306	3.11.1.1			766 T>C
	BBF95_00664	BBA85_02394	6-phosphogluconate dehydrogenase (Decarboxylating)	K00033	1.1.1.44 1.1.1.343			623 A>G
	BBF95_01103	BBA85_02119	Tkt2 protein	K00615	2.2.1.1		1969 C>T	
	BBF95_01189	BBA85_02782	Alpha amylase, catalytic domain protein	K01187	3.2.1.20		390 G>A	
	BBF95_01468	BBA85_03237	Methionine transport protein					1484 G>T
	BBF95_02021	BBA85_02774	Glycosyl hydrolase family 85				858 G>A	
	BBF95_02167	BBA85_00311	Sugar transport protein					581 A>C
Amino acid transport and metabolism	BBF95_01897	BBA85_00550	Methionine synthase				561 C>A	
	BBF95_03033	BBA85_02613	Methonine synthase (Cobalamine-independent),C-terminal domain protein					219 A>G
Lipid metabolism	BBF95_02691	BBA85_02973	Lipase/esterase				773 G>A	
Coenzyme metabolism	BBF95_00838	BBA85_00982	Riboflavin biosynthesis protein RibBA	K14652	4.1.99.12 / 3.5.4.25			817 T>C
	BBF95_03047	BBA85_02002	GTP cyclohydrolase 1	K01495	3.5.4.16		556 C>T	
	BBF95_03069	BBA85_01544	2-dehydropantoate 2-reductase	K00077	1.1.1.169			879 A>C
Inorganic ion transport and metabolism	BBF95_01532	BBA85_03089	ABC transporter, ATP-binding protein	K01990			28 C >T	
	BBF95_02412	BBA85_02307	Nitrate/sulfonate/bicarbonate ABC transporter ATP-binding protein	K02049			67 C>T	
Energy production and conversion	BBF95_01500	BBA85_03055	Isopentenyl-diphosphate delta-isomerase	K01823	5.3.3.2			928 T>A
	BBF95_01581	BBA85_03137	Pyridine nucleotide-disulfide oxidoreductase	K00383	1.8.1.7		625 G>T	
General function prediction only	BBF95_01756	BBA85_02705	Streptomycin 3''-adenylyltransferase				59 T>A	
	BBF95_02087	BBA85_00214	GNAT family acetyltransferase					187 T>C
Secondary metabolites biosynthesis, transport, and catabolism	BBF95_03015	BBA85_02595	Dehydrosqualene desaturase	K10027	1.3.99.26 / 1.3.99.28 / 1.3.99.29 1.3.99.31		1145 C>A	
DNA replication, recombination, repair	BBF95_00026	BBA85_01496	DNA replication protein					536 A>C
	BBF95_02353	BBA85_02253	Putative 3-methyladenine DNA glycosylase	K03652	3.2.2.21		243 G>A	
Defense mechanisms	BBF95_00140	BBA85_02925	Immunity protein PlnL				667 C>T	
	BBF95_00194	BBA85_00864	Beta-lactamase class A	K17836	3.5.2.6		825 C>A	
	BBF95_01291	BBA85_00075	Multidrug ABC transporter, ATP-binding and permease protein	K18891			34 C>T	
	BBF95_01550	BBA85_03106	Chloramphenicol acetyltransferase					269 G>T
Signal transduction mechanisms	BBF95_00250	BBA85_02818	Histidine kinase					1039 T>C
	BBF95_01475	BBA85_03030	Carotenoid biosynthetic protein CrtK	K05770				324 A>T
	BBF95_02170	BBA85_00313	Cell filamentation protein Fic					169 T>C
Transcription	BBF95_00030	BBA85_01500	Cro/CI family transcriptional regulator				257 T>G	
	BBF95_00110	BBA85_02894	Transcription regulator, Rrf2 family				463 G>T	
	BBF95_00944	BBA85_03003	AraC family transcriptional regulator					850 T>C
	BBF95_01129	BBA85_01041	Malolactic regulator, LysR family					744 A>C
Cell envelope biogenesis, outer membrane	BBF95_00127	BBA85_02908	Extracellular protein				647 G>A	
	BBF95_00698	BBA85_00703	Extracellular protein					822 A>G
	BBF95_01257	BBA85_00051	Cell surface protein				739 C>T	
	BBF95_01305	/	Adherence-associated mucus-binding protein,LPXTG-motif cell wall anchor				1108 C>T	
	BBF95_01306	BBA85_02426	Cell surface protein					2635 T>G
	BBF95_01386	BBA85_02992	Predicted membrane protein				508 C>T	
	BBF95_01574	BBA85_03130	Putative membrane protein				1311 C>A	
	BBF95_01640	BBA85_01579	Lpxtg-motif cell wall anchor domain protein					1408 T>C
	BBF95_02026	BBA85_03238	Cell surface SD repeat protein,membrane-anchored			2 T>C		
	BBF95_02171	BBA85_00313	Cell division protein Fic					160 T>C
	BBF95_02506	BBA85_01286	Cell surface protein, LPXTG-motif cell wall anchor					2419 T>C
	BBF95_02660	BBA85_00522	Putative membrane protein					539 A>G; 538 T>C
Mobilome: prophages, transposons, plasmid genes	BBF95_00066	/	Plasmid pLP-ZJ103, complete sequence				4 G>T	
	BBF95_01378	BBA85_00404	Prophage tail length tape-measure protein				3830 C>G	
	BBF95_01381	BBA85_00408	Phage tail protein			1 A>G		
	BBF95_01382	BBA85_00409	Prophage protein, DUF646 family				350 C>G	
	BBF95_01684	BBA85_00017	Prophage P2a protein 53					790 T>G
	BBF95_02031	BBA85_00940	Transposase IS30 (Fragment)			2 T>C		
	BBF95_02748	BBA85_02421	Transposase (Fragment)					408 A>T
Function unknown / Not assigned	BBF95_01316	BBA85_02438	Hypothetical cytosolic protein				421 C>T	
	BBF95_00177	BBA85_02952	Putative uncharacterized protein					215 A>G
	BBF95_00252	BBA85_02820	Putative uncharacterized protein				283 C>T	
	BBF95_00772	/	Putative uncharacterized protein				126 G>A	
	BBF95_00885	BBA85_00160	Putative uncharacterized protein				82 C>T	
	BBF95_01460	/	Putative uncharacterized protein				197 G>A	
	BBF95_01673	BBA85_01098	Addiction module toxin, PemK-like protein					388 T>G
	BBF95_01776	BBA85_02722	DNA-binding protein with HIRAN domain					231 A>G
	BBF95_02280	/	Putative uncharacterized protein				16 C>T	
	BBF95_02605	/	Putative uncharacterized protein			3 G>A	91 C>T	
	BBF95_02627	BBA85_01538	Diguanylate cyclase/phosphodiesterase, GGDEF domain protein					670 T>C
	BBF95_02760	BBA85_01925	ATP-dependent DNA helicase/translocase (Putative)					103 T>G

Supplementary Table 3. Genes exclusively hosted in the genotype O2T60C

GIs	Locus tags	Genes	Encoded proteins	KO	EC number
GI 1	BBA85_00118		FecB protein	K02016	
	BBA85_00119		Iron chelating ABC transporter, ATP-binding protein	K02013	3.6.3.34
	BBA85_00120		Iron chelating ABC transporter, permease protein	K02015	
	BBA85_00121	*ykuN*	Flavodoxin		
	BBA85_00122	*moaE*	Molybdopterin biosynthesis protein, E chain	K03635	2.8.1.12
	BBA85_00123		Molybdopterin converting factor, small subunit	K03636	
	BBA85_00124	*moaA*	Cyclic pyranopterin monophosphate synthase	K03639	4.1.99.22
	BBA85_00125	*narK*	Nitrite extrusion protein	K02575	
	BBA85_00126		N-6 adenine-specific DNA methylase YitW		
	BBA85_00127		XRE family transcriptional regulator		
	BBA85_00128		Putative uncharacterized protein		
	BBA85_00129		Putative uncharacterized protein		
	BBA85_00130		Putative uncharacterized protein		
	BBA85_00131	*nreC*	Two component transcriptional regulator, LuxR family	K07696	
	BBA85_00132	*nreB*	Sensor histidine kinase	K07683	2.7.13.3
	BBA85_00133		NreA	K10851	
	BBA85_00134		Putative uncharacterized protein		
	BBA85_00135	*mobA*	Molybdopterin-guanine dinucleotide biosynthesis protein MobA (Putative)	K03752	
	BBA85_00136	*moaC*	Cyclic pyranopterin monophosphate synthase accessory protein	K03637	4.6.1.17
	BBA85_00137	*mobB*	MobB protein	K03753	
	BBA85_00138	*moeA*	Molybdopterin biosynthesis protein MoeA	K03750	2.10.1.1
	BBA85_00139	*moaB*	Molybdenum cofactor biosynthesis protein B	K03638	
	BBA85_00140	*thiF*	MoeB protein	K21029	2.7.7.80
	BBA85_00141	*narG*	Nitrate reductase, alpha chain	K00370	1.7.5.1 1.7.99.4
	BBA85_00142	*narH*	Nitrate reductase, beta chain	K00371	1.7.5.1 1.7.99.4
	BBA85_00143	*narJ*	Nitrate reductase, delta chain	K00373	
	BBA85_00144	*narI*	Nitrate reductase, gamma chain	K00374	1.7.5.1 1.7.99.4
	BBA85_00148		Cro/CI transcriptional regulator	K07727	
GI 2	BBA85_01811	*rihA*	Inosine-uridine nucleoside N-ribohydrolase	K01250	
	BBA85_01812		Oxidoreductase, medium chain dehydrogenase/reductase family (MDR)		
	BBA85_01813		UPF0303 protein CMPG5300_2915		
	BBA85_01814	*yrbE*	Myo-inositol 2-dehydrogenase-like (Promiscuous)	K00010	1.1.1.18 1.1.1.369
	BBA85_01815	*iolT*	Myo-inositol (And similar sugars) transporter, major facilitator superfamily (MFS)		
	BBA85_01816	*yrbE*	Myo-inositol 2-dehydrogenase-like (Promiscuous)	K00010	1.1.1.18 1.1.1.369
	BBA85_01817	*iolE*	Inosose dehydratase	K03335	4.2.1.44
	BBA85_01818	*iolG*	Inositol 2-dehydrogenase/D-chiro-inositol 3-dehydrogenase	K00010	1.1.1.18 1.1.1.369
	BBA85_01819	*iolX*	Myo-inositol 2-dehydrogenase-like (Promiscuous)	K00010	1.1.1.18 1.1.1.369
	BBA85_01821		Sugar-phosphate aldolase	K01628	4.1.2.17
	BBA85_01822		Putative uncharacterized protein		
	BBA85_01823	*gatC*	Pts36C protein	K02775	
	BBA85_01824		Galactitol PTS, EIIB		
	BBA85_01825		Galactitol PTS, EIIA	K02773	2.7.1.200
	BBA85_01826	*iolR*	Transcription regulator, DeoR family		
	BBA85_01827		Plasmid pLP-ZJ103, complete sequence		
GI 3	BBA85_00355	*cmtB*	PTS family L-ascorbate (L-asc) porter component IIA		
	BBA85_00356	*ppaX*	Gph3 protein		
	BBA85_00357		L-iditol 2-dehydrogenase	K00008	1.1.1.14
	BBA85_00358	*gatC*	Galactitol PTS, EIIC	K02775	
	BBA85_00359	*gatB*	Putative N(Pi)-phosphohistidine--sugar phosphotransferase	K02774	2.7.1.200
	BBA85_00360	*gatA*	Phosphoenolpyruvate-dependent sugar phosphotransferase system, EIIA 2	K02773	2.7.1.200
	BBA85_00361	*glpR*	DeoR family transcriptional regulator	K02444	
	BBA85_00362	*lp_3551*	Probable phosphoketolase		
	BBA85_00363		Transcription regulator		
	BBA85_00364		Putative uncharacterized protein		
	BBA85_00365		Lipase/esterase		
	BBA85_00366	*xynB*	Lipase/esterase (Putative)		
	BBA85_00367	*sglT*	Sodium-coupled N-acetylneuraminate transporter	K03307	
GI 4	BBA85_00391		Putative uncharacterized protein		
	BBA85_00392		Sugar specific permease (Putative)		
	BBA85_00393	*rhaD*	Rhamnulose-1-phosphate aldolase	K01629	4.1.2.19
	BBA85_00394	*rhaA*	L-rhamnose isomerase	K01813	5.3.1.14
	BBA85_00395	*rhaM*	L-rhamnose mutarotase	K03534	
	BBA85_00396	*rhaB*	Rhamnulokinase	K00848	2.7.1.5
	BBA85_00397	*iolF*	Sugar transport protein	K06610	
	BBA85_00398		Transcription regulator		
	BBA85_00399	*ydjF*	Transcription regulator, DeoR family		
GI 5	BBA85_02470	*fhuB*	Ferrichrome ABC transporter, permease protein	K02015	
	BBA85_02471	*fhuG*	FhuG protein		
	BBA85_02472	*feuC*	Iron ABC transporter permease	K02015	
	BBA85_00348	*lytS*	Two-component system, sensor histidine kinase	K07718	2.7.13.3
	BBA85_00371	*nanA*	N-acetylneuraminate lyase	K01639	4.1.3.3
	BBA85_00444		RepB protein		
	BBA85_00445		RepA protein		
	BBA85_00448		RepB protein		
	BBA85_00449		RepA protein		
	BBA85_00462		Putative mucus binding protein		
	BBA85_00858		Putative mucus binding protein		
	BBA85_00922	*mobA*	Mobilization protein MobA		
	BBA85_00925		Replication protein RepB		
	BBA85_00928	*chpB*	MazF family toxin-antitoxin system		
	BBA85_00933		Mobilization protein MobA		
	BBA85_00942	*bin3*	DNA integration/recombination/inversion protein		
	BBA85_00954		Putative mucus binding protein		
	BBA85_01088		Putative resolvase		
	BBA85_01712	*mtlA*	Pts2CB protein	K02799	2.7.1.197
	BBA85_01729		Putative uncharacterized protein (Fragment)		
	BBA85_01774		50S ribosomal protein L34	K02914	

	BBA85_02211	*desR*	Rrp6 protein	K07693	
	BBA85_02212	*yvfT*	Two-component system histidine protein kinase, sensor protein	K07778	2.7.13.3
	BBA85_02216	*guaA*	Acetyltransferase (Putative)	K03827	
	BBA85_02219		Draft genome		
	BBA85_02320		Rep		
	BBA85_02487	*nudC*	Pyrophosphatase (Putative)	K03426	3.6.1.22
	BBA85_02765	*glgE*	Alpha-amylase	K01176	3.2.1.1
	BBA85_02808		CDP-glycerol:poly(Glycerophosphate) glycerophosphotransferase	K09809	
	BBA85_02809		CDP-glycerol:poly(Glycerophosphate) glycerophosphotransferase		
	BBA85_03166		Putative uncharacterized protein (Fragment)		
	BBA85_03189		Type II restriction enzyme, methylase subunit		
	BBA85_03191		Type II restriction enzyme, methylase subunit		
**Mobilome: prophages, transposons, plasmid genes**	BBA85_00405		Prophage protein		
	BBA85_00599		Prophage protein		
	BBA85_00601		Prophage Lp2 protein 26		
	BBA85_00604		Prophage protein		
	BBA85_00624		Cro/CI family phage transcriptional regulator		
	BBA85_00859		Transposase		
	BBA85_00936		Transposase-like protein		
	BBA85_00943		Plasmid pLP-ZJ103, complete sequence		
	BBA85_00948		Transposase		
	BBA85_00949		Transposase, IS4 family		
	BBA85_01074		Transposase		
	BBA85_01083		Plasmid pLP-ZJ103, complete sequence		
	BBA85_01084		Transposase		
	BBA85_01108		Prophage Lp2 protein 26		
	BBA85_01110		Prophage protein		
	BBA85_01116		Prophage protein		
	BBA85_01287		Transposase		
	BBA85_01430		Toxin-antitoxin system, toxin component, PemK/MazF family		
	BBA85_01473		Prophage protein, immunity repressor		
	BBA85_01476		Prophage Lp2 protein 6		
	BBA85_01730		Transposase		
	BBA85_01731		Transposase		
	BBA85_01827		Plasmid pLP-ZJ103, complete sequence		
	BBA85_01828		Transposase		
	BBA85_01952		Plasmid pLP-ZJ103, complete sequence		
	BBA85_01953		Transposase		
	BBA85_01975		Plasmid LBPp5, complete sequence		
	BBA85_02354		Transposase		
	BBA85_02371		Transposase		
	BBA85_02372		Transposase, IS4 family		
	BBA85_02805		Transposase		
	BBA85_02811		Transposase		
	BBA85_02964		Transposase		
	BBA85_02965		Plasmid pLP-ZJ103, complete sequence		
	BBA85_03157		Transposase		
	BBA85_03158		Transposase (Fragment)		
	BBA85_03212		Putative transposase (Putative)		

Supplementary Table 4. Genes exclusively hosted in the genotype S2T10D/S11T3E

	Locus tags				
Gis	S2T10D	S11T3E	Encoded proteins	KO	EC number
GI 6	BBF95_00141	BBA84_02481	Plantaricin biosynthesis protein PlnQ		
	BBF95_00142	BBA84_02482	Three-componet system histidine protein kinase PlnB, sensor protein	K07706	2.7.13.3
	BBF95_00143	BBA84_02483	Three-component response regulator PlnC, activator	K07707	
	BBF95_00144	BBA84_02484	Three-component response regulator PlnD, repressor	K07707	
	BBF95_00145	BBA84_02485	Bacteriocin immunity protein PlnI, membrane-bound protease CAAX family		
	BBF95_00146	BBA84_02486	Bacteriocin peptide PlnF		
	BBF95_00147	BBA84_02487	Bacteriocin peptide PlnE (Putative)		
	BBF95_00148	BBA84_02488	Bacteriocin ABC-transporter, ATP-binding and permease protein PlnG		
	BBF95_00150	BBA84_02489	Bacteriocin ABC-transporter, ATP-binding and permease protein PlnG	K20344	
	BBF95_00151	BBA84_02490	Putative putative accessory factor for ABC-transporter (plnH)		
	BBF95_00152	BBA84_02491	Bacteriocin ABC transporter, accessory factor PlnH	K20345	
GI 7	BBF95_02519	BBA84_01067	Type I restriction-modification system.restriction subunit	K01153	
	BBF95_02520	BBA84_01068	HsdM protein	K03427	
	BBF95_02521	BBA84_01069	Restriction-modification system specificity subunit		
	BBF95_02522	BBA84_01070			
	BBF95_02523	BBA84_01071	Type I restriction modification DNA specificity domain protein (Fragment)		
	BBF95_02524	BBA84_01072	Type I restriction modification DNA specificity domain protein		
GI 8	BBF95_00632	BBA84_00359	Glycosyltransferase. group 2 family protein	K07011	
	BBF95_00633	BBA84_00360	Glycosyl transferase family 2	K07011	
	BBF95_00634	BBA84_00361	UDP-galactopyranose mutase	K01854	
	BBF95_00635	BBA84_00362	Polysaccharide biosynthesis protein		
	BBF95_00636	BBA84_00363	Cps3E protein		
	BBF95_00637	BBA84_00364	Polysaccharide biosynthesis membrane protein		
	BBF95_00638	BBA84_00365	Polysaccharide biosynthesis protein		
GI 9	BBF95_01179	BBA84_00353	UDP-galactopyranose mutase	K01854	
	BBF95_01181	BBA84_00355	Polysaccharide biosynthesis protein		
	BBF95_01182	BBA84_00356	Capsule chain length determinant protein		
	BBF95_01183	BBA84_00357	Polysaccharide biosynthesis protein		
GI 10	BBF95_01848	BBA84_00131	Uncharacterized membrane protein yvbJ		
	BBF95_01847	BBA84_00132	Putative membrane protein		
	BBF95_01846	BBA84_00133	Lipoprotein		
GI 11	BBF95_02607	BBA84_00608	Putative glycosyltransferase TIGR03111		
	BBF95_02608	BBA84_00609	Ica2 protein		
	BBF95_02609	BBA84_00610	Integral membrane protein		
	BBF95_01659	BBA84_00167	Transcription regulator		
	BBF95_01655	BBA84_00168	Formate C-acetyltransferase	K00656	2.3.1.54
	BBF95_00138	BBA84_02478	Putative bacteriocin		
	BBF95_03033	BBA84_00782	Methonine synthase (Cobalamine-independent).C-terminal domain protein		
	BBF95_00084	BBA84_01090	Lactococcin A immunity protein		
	BBF95_00345	BBA84_01719	Oligopeptide ABC transporter substrate binding protein		
	BBF95_02428	BBA84_02313	Putative LtrC		
	BBF95_01404	BBA84_02574	Peptidase E		
	BBF95_03092	BBA84_02701	Putative pyruvate oxidase		
	BBF95_00663	BBA84_00389	6-phosphogluconate dehydrogenase. decarboxylating		
	BBF95_02255	BBA84_01204	Glucose-1-P adenylyltransferase, regulatory subunit		
	BBF95_02167	BBA84_01389	Sugar transport protein		
	BBF95_02708	BBA84_01410	ABC transporter permease		
	BBF95_02709	BBA84_01411	Carbohydrate ABC transporter membrane protein 1, CUT1 family		
	BBF95_02710	BBA84_01412	Sugar ABC transporter substrate-binding protein		
	BBF95_02711	BBA84_01413	Glycosyl hydrolase family 65 central catalytic domain protein		
	BBF95_02718	BBA84_01420	Transport protein, MFS family		
	BBF95_02721	BBA84_01423	Bifunctional protein		
	BBF95_01651	BBA84_01475	Sugar transport protein		
	BBF95_03086	BBA84_01498	Phosphoenolpyruvate-dependent sugar phosphotransferase system EIIABC	K02757	
	BBF95_03087	BBA84_01499	6-phospho-beta-glucosidase	K01223	3.2.1.86
	BBF95_00072	BBA84_01885	Arabinose efflux permease		
	BBF95_02739	BBA84_02302	Drug resistance transporter, MFS superfamily, EmrB/QacA family		
	BBF95_02056	BBA84_02331	Drug resistance transporter, EmrB/QacA subfamily		
	BBF95_00220	BBA84_02344	Permease IIC component	K02761	
	BBF95_00226	BBA84_02350	KdgK protein	K00874	2.7.1.45
	BBF95_00247	BBA84_02371	Transport protein, major facilitator superfamily (MFS)		
	BBF95_02045	BBA84_02713	Glucose-1-phosphate adenylyltransferase, subunit		
	BBF95_00073	BBA84_01886	Polysaccharide pyruvyl transferase		
	BBF95_01408	BBA84_02698	Beta-phosphoglucomutase		
	BBF95_01660	BBA84_00166	Transaldolase		
	BBF95_02572	BBA84_00574	Glycosyltransferase. family 1 (GT1)		
	BBF95_00070	BBA84_01081	Glycosyltransferase		
	BBF95_02128	BBA84_01350	Extracellular protein,gamma-D-glutamate-meso-diaminopimelate muropeptidase		
	BBF95_00318	BBA84_01902	UDP-galactopyranose mutase		
	BBF95_02275	BBA84_01224	ATPase, AAA family		
	BBF95_02786	BBA84_01575	Glycosyltransferase, group 2 family protein		
	BBF95_02440	BBA84_02325	Copy number control protein		
	BBF95_02740	BBA84_02301	Ferrochelatase		
	BBF95_02542	BBA84_02686	Methyltransferase family protein		
	BBF95_00839	BBA84_02911	Riboflavin biosynthesis protein RibBA		
	BBF95_00346	BBA84_01720	Proline iminopeptidase		
	BBF95_00347	BBA84_01721	Proline iminopeptidase		
	BBF95_01407	BBA84_02697	Cof-like hydrolase		
	BBF95_00655	BBA84_00382	Extracellular protein. beta-lactamase family		
	BBF95_00656	BBA84_00383	Serine-type D-Ala-D-Ala carboxypeptidase		
	BBF95_01550	BBA84_01955	Chloramphenicol acetyltransferase		
	BBF95_01609	BBA84_02013	Mrr restriction system protein	K07448	
	BBF95_02741	BBA84_02300	Antimicrobial peptide ABC transporter, ATPase component		
	BBF95_02938	BBA84_00827	ICEBs1 integrase		
	BBF95_02503	BBA84_01051	Superfamily I DNA/RNA helicase		
	BBF95_02504	BBA84_01052	Superfamily I DNA/RNA helicase		
	BBF95_02042	BBA84_01484	RepB protein		
	BBF95_02760	BBA84_01548	ATP-dependent DNA helicase/translocase (Putative)		
	BBF95_00079	BBA84_01892	DNA polymerase III subunit epsilon		
	BBF95_02358	BBA84_02126	Integrase		
	BBF95_02032	BBA84_02292	Site-specific recombinase, DNA invertase Pin-related protein		
	BBF95_02736	BBA84_02305	ImpB/MucB/SamB family protein (Fragment)		
	BBF95_00079	BBA84_02324	DNA polymerase III subunit epsilon		
	BBF95_01658	BBA84_01480	Formate C-acetyltransferase		
	BBF95_02423	BBA84_02287	Membrane protein, YkoY family		
	BBF95_02738	BBA84_02303	Heme peroxidase HemQ, essential component of heme biosynthetic pathway in Gram-positive bacteria		
	BBF95_02645	BBA84_00883	Signal peptidase I	K03100	3.4.21.89
	BBF95_00643	BBA84_00370	Cell wall surface anchor family protein		
	BBF95_00966	BBA84_00487	Integral membrane protein		
	BBF95_00741	BBA84_00507	Integral membrane protein		
	BBF95_00677	BBA84_00611	Extracellular protein		
	BBF95_00697	BBA84_00631	Cell surface protein. CscB family		
	BBF95_02968	BBA84_00718	Putative membrane protein		
	BBF95_03041	BBA84_00790	Putative membrane protein		
	BBF95_00069	BBA84_01082	Integral membrane protein		
	BBF95_02713	BBA84_01415	Membrane protein		
	BBF95_01325	BBA84_01461	Cell surface protein		
	BBF95_02747	BBA84_01487	Integral membrane protein		
	BBF95_00046	BBA84_01502	Membrane protein		
	BBF95_00047	BBA84_01503	Membrane protein		
	BBF95_02783	BBA84_01572	Putative uncharacterized protein		
	BBF95_02784	BBA84_01573	Putative membrane protein		
	BBF95_02613	BBA84_01870	Cell surface protein, LPXTG-motif cell wall anchor		
	BBF95_00075	BBA84_01888	Membrane protein		
	BBF95_01545	BBA84_01950	Membrane protein		
	BBF95_02356	BBA84_02124	Lipoprotein		
	BBF95_00046	BBA84_02183	Membrane protein		
	BBF95_00047	BBA84_02184	Membrane protein		
	BBF95_02029	BBA84_02295	Putative membrane protein		
	BBF95_02742	BBA84_02299	Sensory box protein		
	BBF95_00005	BBA84_02328	Membrane protein		
	BBF95_00125	BBA84_02465	Extracellular protein, lysine-rich		
	BBF95_00126	BBA84_02466	Lmo0142 protein		
	BBF95_00127	BBA84_02467	Extracellular protein		
	BBF95_01402	BBA84_02572	Membrane protein		
	BBF95_02538	BBA84_02682	Membrane protein		
	BBF95_00831	BBA84_02903	Lactobacilli aggregation promoting factor		
	BBF95_01782	BBA84_01804	Purine nucleoside phosphorylase		
	BBF95_01653	BBA84_01477	Formate acetyltransferase activating enzyme	K04069	
	BBF95_01716	BBA84_01738	Peptidase, S8/S53 family		
	BBF95_02535	BBA84_02679	Glutathione S-transferase		
	BBF95_02618	BBA84_01865	Cytochrome P450		
	BBF95_02696	BBA84_00934	Diguanylate cyclase/phosphodiesterase. GGDEF domain protein		
	BBF95_00249	BBA84_02373	Histidine kinase		
	BBF95_02628	BBA84_01441	ATP phosphoribosyltransferase		
	BBF95_00967	BBA84_00488	Transcriptional regulator		
	BBF95_00745	BBA84_00511	Transcriptional regulator. MerR family		
	BBF95_03040	BBA84_00789	XRE family transcriptional regulator		
	BBF95_02707	BBA84_01409	Transcriptional regulator, LacI family		
	BBF95_03085	BBA84_01497	BglG2 protein		
	BBF95_02417	BBA84_02289	Mn-dependent transcriptional regulator MntR		
	BBF95_00219	BBA84_02343	Transcriptional regulator, GntR family		
	BBF95_00248	BBA84_02372	Transcription regulator		
	BBF95_00465	BBA84_02670	Sugar-binding domain protein		
	BBF95_01652	BBA84_01476	Bifunctional protein		
	BBF95_02533	BBA84_02708	Stress-responsive transcriptional regulator PspC		
	BBF95_01278	BBA84_03088	Transcriptional regulator, LysR family		
	BBF95_00224	BBA84_02348	L-seryl-tRNA(Sec) selenium transferase-related protein, pyridoxal phosphate-dependent	K01042	2.9.1.1
	BBF95_02929	BBA84_00818	Predicted protein		
	BBF95_02937	BBA84_00826	unknown		
	BBF95_02532	BBA84_01080	Fumarate reductase, flavoprotein subunit		
	BBF95_02276	BBA84_01225	unknown		
	BBF95_02170	BBA84_01393	Cell filamentation protein Fic		
	BBF95_02171	BBA84_01394	Cell division protein Fic		
	BBF95_02712	BBA84_01414	unknown		
	BBF95_02040	BBA84_01483	Copy-number control protein		
	BBF95_00048	BBA84_01504	ABC transporter ATP-binding protein		

	BBF95_02785	BBA84_01574	Putative uncharacterized protein		
	BBF95_02787	BBA84_01576	Glycosyl transferase		
	BBF95_02788	BBA84_01577	GGDEF domain protein		
	BBF95_01776	BBA84_01798	DNA-binding protein with HIRAN domain		
	BBF95_01803	BBA84_01826	unknown		
	BBF95_00071	BBA84_01884	ThiJ/PfpI family protein		
	BBF95_00048	BBA84_02185	ABC transporter ATP-binding protein		
	BBF95_02429	BBA84_02314	Lactobacillus brevis strain TMW 1,313 Scaffold_2_TMW1313, whole genome shotgun sequence		
	BBF95_02441	BBA84_02326	Orf5 protein		
	BBF95_00222	BBA84_02346	PTS-associated protein		
	BBF95_00223	BBA84_02347	Metallo-dependent hydrolase, subgroup B	K01465	3.5.2.3
	BBF95_00225	BBA84_02349	2-dehydro-3-deoxyphosphooctonate aldolase		
	BBF95_00189	BBA84_02528	Extracellular protein		
	BBF95_01399	BBA84_02569	Hypothetical cytosolic protein		
	BBF95_01400	BBA84_02570	Transglycosylase associated protein family		
	BBF95_01401	BBA84_02571	Stress response regulator Gls24		
	BBF95_01403	BBA84_02573	Trypsin-like serine protease		
	BBF95_02537	BBA84_02681	Transcriptional regulator		
	BBF95_02539	BBA84_02683	Variovorax paradoxus strain MEDvA23 contig_22, whole genome shotgun sequence		
	BBF95_02540	BBA84_02684	Metallo-beta-lactamase domain protein		
	BBF95_01980	BBA84_02694	Barstar (Barnase inhibitor)		
	BBF95_00075	BBA84_02705	Membrane protein		
**Mobilome: prophages, transposons, plasmid genes**	BBF95_01657	BBA84_00169	Transposase		
	BBF95_00395	BBA84_00170	Transposase		
	BBF95_01686	BBA84_00412	Prophage P2a protein 53		
	BBF95_01816	BBA84_00956	Prophage P2a protein 47		
	BBF95_01817	BBA84_00957	Prophage P2a protein 46		
	BBF95_01818	BBA84_00958	Prophage P2a protein 45		
	BBF95_01819	BBA84_00959	Prophage P2a protein 45		
	BBF95_01820	BBA84_00960	Prophage P2a protein 44		
	BBF95_01821	BBA84_00961	Prophage P2a protein 43		
	BBF95_01822	BBA84_00962	Prophage P1 protein 44		
	BBF95_01823	BBA84_00963	Prophage P2a protein 41		
	BBF95_01824	BBA84_00964	Prophage P2a protein 40		
	BBF95_01825	BBA84_00965	Prophage P2a protein 39		
	BBF95_01983	BBA84_00967	Prophage P2a protein 37		
	BBF95_01985	BBA84_00970	Phage terminase. small subunit		
	BBF95_01986	BBA84_00971	Enterococcus gallinarum strain A6981 Contig019. whole genome shotgun sequence		
	BBF95_01988	BBA84_00973	Prophage P2a protein 33		
	BBF95_01991	BBA84_00977	Group-specific protein		
	BBF95_01995	BBA84_00981	Hypothetical phage protein		
	BBF95_01339	BBA84_00982	Prophage protein		
	BBF95_02000	BBA84_00985	Prophage Lp2 protein 23		
	BBF95_02001	BBA84_00986	Bacteriophage antirepressor		
	BBF95_02004	BBA84_00989	Prophage Lp1 protein 19		
	BBF95_02005	BBA84_00990	Recombinational DNA repair protein RecT (Prophage associated)	K07455	
	BBF95_02006	BBA84_00991	Prophage Lp1 protein 17		
	BBF95_02011	BBA84_00996	Prophage Lp1 protein 9		
	BBF95_02012	BBA84_00997	Prophage Lp1 protein 8		
	BBF95_02033	BBA84_01001	Prophage protein	K15051	
	BBF95_00025	BBA84_01024	Prophage Lp3 protein 8. helicase		
	BBF95_02485	BBA84_01230	Abi family protein		
	BBF95_02487	BBA84_01232	Holin		
	BBF95_02489	BBA84_01234	XkdX family protein		
	BBF95_02491	BBA84_01236	Prophage protein, minor tail protein		
	BBF95_02492	BBA84_01237	Prophage protein		
	BBF95_02493	BBA84_01238	Prophage P2a protein 51		
	BBF95_02494	BBA84_01239	Prophage P2a protein 50		
	BBF95_01456	BBA84_01253	Lactobacillus brevis strain TMW 1,313 Scaffold_1_TMW1313, whole genome shotgun sequence		
	BBF95_02732	BBA84_01433	Transposase		
	BBF95_00049	BBA84_01505	Putative transposase		
	BBF95_00050	BBA84_01506	Transposase tnp4		
	BBF95_02617	BBA84_01866	Transposase		
	BBF95_02616	BBA84_01867	Transposase subunit B (Fragment)		
	BBF95_02615	BBA84_01868	Transposase		
	BBF95_00049	BBA84_02186	Putative transposase		
	BBF95_02031	BBA84_02293	Transposase IS30 (Fragment)		
	BBF95_02028	BBA84_02296	Transposase		
	BBF95_02432	BBA84_02317	Plasmid LBPp4, complete sequence		
	BBF95_02435	BBA84_02320	Plasmid LBPp4, complete sequence		
	BBF95_02436	BBA84_02321	Lactobacillus sakei strain KCA311 plasmid pKCA54, complete sequence		
	BBF95_00004	BBA84_02327	Transposase, IS30 family		
	BBF95_00006	BBA84_02329	Plasmid LBPp1, complete sequence		
	BBF95_02057	BBA84_02332	Plasmid LBPp1, complete sequence		
	BBF95_01398	BBA84_02568	Plasmid LBPp1, complete sequence		
	BBF95_00468	BBA84_02673	Transposase		
	BBF95_00469	BBA84_02674	Plasmid pW56, complete sequence		
	BBF95_02534	BBA84_02707	Plasmid LBPp4, complete sequence		
	BBF95_01370	BBA84_02760	Prophage P2a protein 55		
	BBF95_01371	BBA84_02761	Prophage P2a protein 54		
	BBF95_01329	BBA84_02779	Phage terminase-like protein, large subunit		
	BBF95_01332	BBA84_02782	Endonuclease		
	BBF95_01339	BBA84_02789	Prophage protein		
	BBF95_01340	BBA84_02790	Prophage protein		
	BBF95_01342	BBA84_02792	Crossover junction endodeoxyribonuclease RusA		
	BBF95_01353	BBA84_02803	Prophage protein		
	BBF95_01354	BBA84_02804	Prophage Lp3 protein 4		
	BBF95_01355	BBA84_02805	CRISPR-associated protein Cas2		
	BBF95_01357	BBA84_02807	Strain AB04 contig000007, whole genome shotgun sequence		
	BBF95_01359	BBA84_02809	Prophage protein		
	BBF95_01361	BBA84_02811	Putative uncharacterized protein ps106		
	BBF95_01364	BBA84_02813	Weissella cibaria strain AB3b contig000011, whole genome shotgun sequence		
	BBF95_01365	BBA84_02814	Capsule polysaccharide export inner-membrane protein BexB		
	BBF95_02930	BBA84_00819	Prophage protein		
	BBF95_02931	BBA84_00820	Pathogenicity island protein		
	BBF95_02932	BBA84_00821	Prophage Lp3 protein 8. helicase		
	BBF95_02936	BBA84_00825	Phage protein		
	BBF95_00067	BBA84_01084	Uncultured bacterium extrachromosomal DNA RGI01644		
	BBF95_00083	BBA84_01089	Phage Integrase		
	BBF95_00068	BBA84_01083	Integrase core domain protein		
	BBF95_00085	BBA84_01091	Putative integrase/recombinase		
	BBF95_01717	BBA84_01739	Draft genome		
	BBF95_01396	BBA84_02307	Lactobacillus brevis strain TMW 1,313 Scaffold_6_TMW1313, whole genome shotgun sequence		
	BBF95_01395	BBA84_02308	Lactobacillus brevis strain TMW 1,313 Scaffold_2_TMW1313, whole genome shotgun sequence		
	BBF95_01394	BBA84_02309	DNA-directed RNA polymerase beta subunit		
	BBF95_00076	BBA84_01889	Phage capsid and scaffold		
	BBF95_03091	BBA84_02702	Phage-related minor capsid protein		
	BBF95_00076	BBA84_02703	Phage capsid and scaffold		
	BBF95_00080	BBA84_01893	Prophage Lp1 protein 60 (Fragment)		
	BBF95_02030	BBA84_02294	ABC exporter ATP-binding subunit, DevA family		
	BBF95_01397	BBA84_02306	Lipoyltransferase and lipoate-protein ligase		
	BBF95_01405	BBA84_02575	Weissella cibaria strain AB3b contig000085, whole genome shotgun sequence		
	BBF95_01406	BBA84_02696	Weissella cibaria strain AB3b contig000085, whole genome shotgun sequence		

Supplementary Table 5. Glucose, lactic acid, acetic acid and butyric acid contents (mM), variation of pH and growth capability (calculated following the formula: Log CFU mL-1after 24 h – Log CFU mL-1initial inoculums) recorded during the fermentation of four different media inoculated with 8.0 ± 0.2 Log CFU of L. plantarum O2T60C and S2T60C, and incubated for 48 h at 37 °C. Fermentation were carried out in (A) Phosphate Buffer Saline (PBS) with 0.45 % of glucose; (B) Dulbecco's Modified Eagle's Medium (DMEM) supplemented with 2 mM of L-glutamine and 0.45 % of glucose; (C) Dulbecco's Modified Eagle's Medium (DMEM) supplemented with 6 mM of L-glutamine and 0.45 % of glucose; (D) modified MRS (mMRS) supplemented with 0.45 % of glucose. Significant differences between the two strains O2T60C and S2T60D are highlighted with asterisk (T-test or Kolmogorov–Smirnov test; *= p <0.05; **= p <0.01; ***= p <0.001).

		mM														
		**Glucose**			**Lactic acid**			**Acetic acid**			**Butyric acid**		**Δ Log CFU**		**pH**		
**Media**	**Hours**	**O2T60C**	**S2T10D**		**O2T60C**	**S2T10D**		**O2T60C**	**S2T10D**		**O2T60C**	**S2T10D**	**O2T60C**	**S2T10D**	**O2T60C**	**S2T10D**	
**A**	0	27.48 ± 0.18	27.48 ± 0.18		/	/		/	/		/	/	/	/	7.54 ± 0.09	7.54 ± 0.09	
	4	23.33 ± 0.10	23.42 ± 0.22		5.97 ± 0.04	4.71 ± 0.11		0.08 ± 0.01	0.08 ± 0.01		n.d.	n.d.	0.10 ± 0.04	0.06 ± 0.05	6.54 ± 0.03	6.57 ± 0.08	
**(PBS)**	8	22.29 ± 0.08	22.46 ± 0.27		9.46 ± 0.31	10.15 ± 0.81		0.12 ± 0.01	0.24 ± 0.10		n.d.	n.d.	-0.13 ± 0.08	0.07 ± 0.06	5.70 ± 0.28	5.76 ± 0.32	
	24	17.02 ± 1.02	17.55 ± 0.83		18.54 ± 1.49	19.15 ± 1.09		0.41 ± 0.02	0.39 ± 0.02		n.d.	n.d.	-0.16 ± 0.11	-0.08 ± 0.03	3.82 ± 0.10	3.74 ± 0.16	
	48	10.70 ± 1.17	9.61 ± 0.81		26.33 ± 1.50	30.75 ± 2.84		0.72 ± 0.05	0.78 ± 0.07		n.d.	n.d.	-0.82 ± 0.23	-0.35 ± 0.11	3.49 ± 0.12	3.48 ± 0.32	
**B**	0	27.48 ± 0.18	27.48 ± 0.18		/	/		0.15 ± 0.01	0.15 ± 0.01		/	/	0.00 ± 0.00	0.00 ± 0.00	8.37 ± 0.07	8.37 ± 0.07	
	4	23.26 ± 0.15	24.17 ± 0.10		7.92 ± 0.03	7.98 ± 0.49		0.25 ± 0.01	0.26 ± 0.02		n.d.	n.d.	0.10 ± 0.01	0.10 ± 0.10	7.53 ± 0.00	7.49 ± 0.06	
**DMEM (2 mM**	8	17.39 ± 0.79	20.27 ± 0.58		20.96 ± 0.56	16.70 ± 1.60		0.35 ± 0.09	0.24 ± 0.12		n.d.	n.d.	0.09 ± 0.11	0.09 ± 0.03	7.34 ± 0.11	7.26 ± 0.06	
**of Gln)**	24	n.d.	n.d.		45.15 ± 0.16	44.74 ± 0.16		1.95 ± 0.34	0.99 ± 0.04		n.d.	n.d.	0.41 ± 0.08	0.23 ± 0.05	4.73 ± 0.02	4.78 ± 0.06	
	48	n.d.	n.d.		46.32 ± 1.13	45.12 ± 0.75		1.78 ± 0.36	2.49 ± 0.10		n.d.	n.d.	-0.11 ± 0.15	-0.54 ± 0.16	4.77 ± 0.05	4.76 ± 0.04	
**C**	0	27.48 ± 0.18	27.48 ± 0.18		/	/		/	/		/	/	0.00 ± 0.00	0.00 ± 0.00	7.92 ± 0.00	7.92 ± 0.00	
	4	23.54 ± 0.36	22.62 ± 0.60		6.34 ± 0.32	8.04 ± 1.01		0.14 ± 0.00	0.15 ± 0.01		**n.d.**	**0.52 ± 0.16**	0.02 ± 0.02	0.06 ± 0.11	7.47 ± 0.08	7.42 ± 0.15	
**DMEM (6 mM**	8	**22.08 ± 0.13**	**17.15 ± 1.16**	*	10.24 ± 0.23	17.95 ± 4.84		0.23 ± 0.04	0.32 ± 0.08		**n.d.**	**1.43 ± 0.44**	0.07 ± 0.07	0.17 ± 0.13	7.50 ± 0.08	7.02 ± 0.17	
**of Gln)**	24	**13.35 ± 0.28**	**n.d.**		**22.96 ± 1.24**	**38.03 ± 1.90**	***	0.46 ± 0.02	0.70 ± 0.07		**n.d.**	**3.07 ± 0.33**	-0.01 ± 0.09	0.09 ± 0.12	**6.74 ± 0.07**	**4.64 ± 0.23**	***
	48	n.d.	n.d.		39.88 ± 2.52	40.89 ± 0.37		**0.86 ± 0.06**	**1.72 ± 0.06**	***	**n.d.**	**3.55 ± 0.23**	-0.21 ± 0.06	-0.57 ± 0.49	**4.59 ± 0.08**	**4.38 ± 0.01**	*
**D**	0	27.48 ± 0.18	27.48 ± 0.18		/	/		6.89 ± 0.19	6.89 ± 0.19		/	/	0.00 ± 0.00	0.00 ± 0.00	6.41 ± 0.03	6.41 ± 0.03	
	4	n.d.	n.d.		52.04 ± 0.21	47.20 ± 0.48		8.84 ± 0.40	8.94 ± 0.25		n.d.	n.d.	0.86 ± 0.11	0.88 ± 0.14	**4.26 ± 0.00**	**4.34 ± 0.02**	*
	8	n.d.	n.d.		57.92 ± 0.40	52.73 ± 3.14		13.99 ± 0.11	12.00 ± 2.71		n.d.	n.d.	0.82 ± 0.02	0.95 ± 0.05	4.30 ± 0.03	4.27 ± 0.02	
**mMRS**	24	n.d.	n.d.		57.73 ± 0.18	56.37 ± 0.42		**20.13 ± 0.29**	**22.34 ± 0.22**	*	n.d.	n.d.	0.05 ± 0.25	-0.50 ± 0.08	4.26 ± 0.03	4.30 ± 0.05	
	48	n.d.	n.d.		58.02 ± 0.53	56.03 ± 0.32		21.29 ± 0.24	22.88 ± 0.27		n.d.	n.d.	-1.23 ± 0.17	-1.01 ± 0.04	4.29 ± 0.04	4.28 ± 0.04	

Supplementary Table 6. Complete list of reactions and genes (L. plantarum S2T10D, L. plantarum O2T60C) involved in glutamine uptake and glutamine/glutamate metabolism of L. plantarum; genes nomenclature is based on the reference genome WCFS1.

		Locus tags						
	**Genes WCFS1**	**S2**	**O2**	**Annotation**	**EC by kass**	**nSNPs**	**Provean**	**Reactions no.**
	glnP	BBF95_02825	BBA85_01180	Glutamine ABC transporter, substrate binding and permease protein		2		
	glnQ1	BBF95_02826	BBA85_01181	ABC-type polar amino acid transport system, ATPase component				
	glnQ2	BBF95_02893	BBA85_01244	ABC-type polar amino acid transport system, ATPase component		1	P231S (-7.602)	
	glnH1	BBF95_02894	BBA85_01245	Glutamine ABC transporter, substrate binding protein				
	glnM	BBF95_02895	BBA85_01246	Glutamine ABC transporter, permease protein				
ABC transporter	*glnP1*	BBF95_02896	BBA85_01247	Glutamine ABC transporter, permease protein				
	glnPH2	BBF95_02581	BBA85_00659	Glutamine ABC transporter, substrate binding and permease protein				
	glnQ3	BBF95_02580	BBA85_00658	ABC-type polar amino acid transport system, ATPase component				
	glnP2	BBF95_00438	BBA85_01355	Glutamine ABC transporter, permease protein				
	glnQ4	BBF95_00437	BBA85_01354	ABC-type polar amino acid transport system, ATPase component		1		
	glnH2	BBF95_00436	BBA85_01353	Glutamine/histidine ABC transporter, substrate binding protein				
	glmS1	BBF95_02844	BBA85_01198	Glutamine--fructose-6-phosphate aminotransferase [isomerizing]	2.6.1.16			R00768
	glmS2	BBF95_02069	BBA85_00196	Glutamine--fructose-6-phosphate transaminase (Isomerizing)	2.6.1.16	1		R00768
	nagB	BBF95_01219	BBA85_01709	Glucosamine-6-phosphate deaminase	3.5.99.6			R00765
Central glutamine/glutamate metabolism	*glnR*	BBF95_00573	BBA85_01621	Glutamine synthetase repressor				
	glnA	BBF95_00574	BBA85_01622	Glutamine synthetase	6.3.1.2			R00253
	gdh	BBF95_01174	BBA85_02355	Glutamate dehydrogenase	1.4.1.4	10	D408A (-2.617)	R00248
	gadB	BBF95_02127	BBA85_00266	Glutamate decarboxylase	4.1.1.15	3	V167A (-3.806)	R00261
	purF	BBF95_01902	BBA85_00555	Amidophosphoribosyltransferase	2.4.2.14	1		R01072
Purine metabolism	*purM*	BBF95_01901	BBA85_00554	Phosphoribosylformylglycinamidine cyclo-ligase	6.3.3.1	6	P303S (-4.064)	R04463
	guaA	BBF95_02500	BBA85_01271	GMP synthase [glutamine-hydrolyzing]	6.3.5.2	1		R01231
	pyrG	BBF95_00203	BBA85_00873	CTP synthase	6.3.4.2			R00573
	carB	BBF95_00337	BBA85_00897	Carbamoyl-phosphate synthase large chain	6.3.5.5	9		R00256/R00575
	carA	BBF95_00338	BBA85_00898	Carbamoyl-phosphate synthase small chain	6.3.5.5	4		R00256/R00575
Pyrimidine, arginine metabolism	*pyrAA2*	BBF95_01543	BBA85_03099	Carbamoyl-phosphate synthase small chain	6.3.5.5	1		R00256/R00575
	pyrAB	BBF95_01885	BBA85_02650	Carbamoyl-phosphate synthase large chain	6.3.5.5	2		R00256/R00575
	pyrAA	BBF95_01886	BBA85_02651	Carbamoyl-phosphate synthase small chain	6.3.5.5	1		R00256/R00575
	argJ	BBF95_00340	BBA85_00500	Arginine biosynthesis bifunctional protein ArgJ	2.3.1.35 2.3.1.1	1		R00259
	gatC	BBF95_01152	BBA85_01063	Aspartyl/glutamyl-tRNA(Asn/Gln) amidotransferase subunit C	6.3.5.6 6.3.5.7	1		R03905
Aminoacyl-tRNA biosynthesis	*gatB*	BBF95_01154	BBA85_01065	Aspartyl/glutamyl-tRNA(Asn/Gln) amidotransferase subunit B	6.3.5.6 6.3.5.7			R03905
	gltX	BBF95_00520	BBA85_01872	Glutamate--tRNA ligase	6.1.1.24			R03651
Arginine metabolism	*proB*	BBF95_02249	BBA85_01760	Glutamate 5-kinase	2.7.2.11	1		R00239
	argD	BBF95_00342	BBA85_00902	Acetylornithine aminotransferase	2.6.1.11	3		R02283
Aspartate metabolism	*asnB1*	BBF95_00420	BBA85_02037	Asparagine synthase (Glutamine-hydrolysing)	6.3.5.4	2		R00578
	asnB2	BBF95_01231	BBA85_02450	Asparagine synthase (Glutamine-hydrolysing)	6.3.5.4	5	D37Y (-6.960)	R00578
Folate metabolism	*folC1*	BBF95_00445	BBA85_01362	Folylpolyglutamate synthase	6.3.2.12 6.3.2.17	4	A347T (-2.867); G14D (-5.278)	R00942
	folC2	BBF95_03046	BBA85_02001	Folylpolyglutamate synthase / dihydrofolate synthase	6.3.2.12 6.3.2.17	3		R00942
Glutathione metabolism	*gshAB*	BBF95_00448	BBA85_01365	Glutamate--cysteine ligase	6.3.2.2	1		R00894
	gshAB	BBF95_00466	BBA85_01381	GshAB protein	6.3.2.2	7		R10993
Gly/Ser/Thr metabolism	*serC*	BBF95_01201	BBA85_02794	Phosphoserine aminotransferase	2.6.1.52			R05085
Histidine biosynthesis	*hisF*	BBF95_00733	BBA85_01559	Imidazole glycerol phosphate synthase subunit HisF	4.1.3.-			R04558
	hisH	BBF95_00735	BBA85_01561	Imidazole glycerol phosphate synthase subunit HisH	2.4.2.-	2		R04558
Peptidoglican biosythesis	*murD*	BBF95_00719	BBA85_00725	UDP-N-acetylmuramoylalanine--D-glutamate ligase	6.3.2.9	2		R02783
	murI	BBF95_01004	BBA85_01322	Glutamate racemase	5.1.1.3			R00260
	trpE	BBF95_00928	BBA85_01687	TrpE protein	4.1.3.27	9		R00986
Phe/Tyr/Trp metabolism	*trpGD*	BBF95_00929	BBA85_01688	Anthranilate synthase, component II	4.1.3.27			R00986
	hisC	BBF95_00730	BBA85_01556	Histidinol-phosphate aminotransferase	2.6.1.9	2		R03243
Amino acid metabolisms	*ilvE*	BBF95_02478	BBA85_01422	Branched-chain-amino-acid aminotransferase	2.6.1.42			R02199

Reactions
L-Glutamine + D-Fructose 6-phosphate <=> L-Glutamate + D-Glucosamine 6-phosphate
L-Glutamine + D-Fructose 6-phosphate => L-Glutamate + D-Glucosamine 6-phosphate
D-Glucosamine 6-phosphate + H2O => D-Fructose 6-phosphate + Ammonia
ATP + L-Glutamate + Ammonia => ADP + Orthophosphate + L-Glutamine
L-Glutamate + NADP+ + H2O <=> 2-Oxoglutarate + Ammonia + NADPH + H+
L-Glutamate => 4-Aminobutanoate + CO2
L-Glutamine + 5-Phospho-alpha-D-ribose 1-diphosphate + H2O <=> 5-Phosphoribosylamine + Diphosphate + L-Glutamate
ATP + 5'-Phosphoribosyl-N-formylglycinamide + L-Glutamine + H2O => ADP + Orthophosphate + 2-(Formamido)-N1-(5'-phosphoribosyl)acetamidine + L-Glutamate
ATP + Xanthosine 5'-phosphate + L-Glutamine + H2O => AMP + Diphosphate + GMP + L-Glutamate
ATP + UTP + L-Glutamine + H2O => ADP + Orthophosphate + CTP + L-Glutamate
L-Glutamine + H2O => L-Glutamate + Ammonia/ATP + L-Glutamine + HCO3- + H2O => 2 ADP + Orthophosphate + L-Glutamate + Carbamoyl phosphate
L-Glutamine + H2O => L-Glutamate + Ammonia/ATP + L-Glutamine + HCO3- + H2O => 2 ADP + Orthophosphate + L-Glutamate + Carbamoyl phosphate
L-Glutamine + H2O => L-Glutamate + Ammonia/ATP + L-Glutamine + HCO3- + H2O => 2 ADP + Orthophosphate + L-Glutamate + Carbamoyl phosphate
L-Glutamine + H2O => L-Glutamate + Ammonia/ATP + L-Glutamine + HCO3- + H2O => 2 ADP + Orthophosphate + L-Glutamate + Carbamoyl phosphate
L-Glutamine + H2O => L-Glutamate + Ammonia/ATP + L-Glutamine + HCO3- + H2O => 2 ADP + Orthophosphate + L-Glutamate + Carbamoyl phosphate
Acetyl-CoA + L-Glutamate **=>** CoA + N-Acetyl-L-glutamate
Glutaminyl-tRNA + L-Glutamate + Orthophosphate + ADP => L-Glutamyl-tRNA(Gln) + L-Glutamine + ATP + H2O
Glutaminyl-tRNA + L-Glutamate + Orthophosphate + ADP => L-Glutamyl-tRNA(Gln) + L-Glutamine + ATP + H2O
ATP + L-Glutamate + tRNA(Gln) => AMP + Diphosphate + L-Glutamyl-tRNA(Gln)
ATP + L-Glutamate <=> ADP + L-Glutamyl 5-phosphate
N-Acetylornithine + 2-Oxoglutarate <= N-Acetyl-L-glutamate 5-semialdehyde + L-Glutamate
ATP + L-Aspartate + L-Glutamine + H2O <=> AMP + Diphosphate + L-Asparagine + L-Glutamate
ATP + L-Aspartate + L-Glutamine + H2O <=> AMP + Diphosphate + L-Asparagine + L-Glutamate
ATP + Tetrahydrofolate + L-Glutamate => ADP + Orthophosphate + THF-L-glutamate
ATP + Tetrahydrofolate + L-Glutamate => ADP + Orthophosphate + THF-L-glutamate
ATP + L-Glutamate + L-Cysteine <=> ADP + Orthophosphate + gamma-L-Glutamyl-L-cysteine
ATP + L-Glutamate + (S)-2-Aminobutanoate <=> ADP + Orthophosphate + gamma-L-Glutamyl-L-2-aminobutyrate
O-Phospho-4-hydroxy-L-threonine + 2-Oxoglutarate <=> 2-Oxo-3-hydroxy-4-phosphobutanoate + L-Glutamate
C04916 + L-Glutamine => C04666 + C04677 + L-Glutamate
C04916 + L-Glutamine => C04666 + C04677 + L-Glutamate
ATP + UDP-N-acetylmuramoyl-L-alanine + D-Glutamate <=> ADP + Orthophosphate + UDP-N-acetylmuramoyl-L-alanyl-D-glutamate
L-Glutamate <=> D-Glutamate
Chorismate + L-Glutamine => Anthranilate + Pyruvate + L-Glutamate
Chorismate + L-Glutamine => Anthranilate + Pyruvate + L-Glutamate
L-Histidinol phosphate + 2-Oxoglutarate <=> 3-(Imidazol-4-yl)-2-oxopropyl phosphate + L-Glutamate
L-Isoleucine + 2-Oxoglutarate <=> (S)-3-Methyl-2-oxopentanoic acid + L-Glutamate
